# Comparative study of flow rate- and material-dependent human plasma protein adsorption on oxygenator membranes and heat exchanger materials

**DOI:** 10.3389/fcvm.2025.1578538

**Published:** 2025-06-17

**Authors:** Katharina Große-Berkenbusch, Meltem Avci-Adali, Patrick Cahalan, Linda Cahalan, Ana Velic, Boris Maček, Christian Schlensak, Hans Peter Wendel, Sandra Stoppelkamp

**Affiliations:** ^1^ Clinical Research Laboratory, Department of Thoracic and Cardiovascular Surgery, University Hospital Tübingen, Tübingen University, Tübingen, Germany; ^2^ Ension Inc., Butler, PA, United States; ^3^ Proteome Center Tübingen, Interfaculty Institute for Cell Biology, University of Tübingen, Tübingen, Germany

**Keywords:** ECMO, hollow fiber membrane, PMP, PET, heparin-coating, plasma protein adsorption, hemocompatibility

## Abstract

Artificial lungs support patients with acute or chronic lung diseases. However, complications such as the activation of blood components leading to thrombosis and inflammation limit their long-term applicability. The systematic characterization of protein adhesion events on different material parts of the oxygenators at different flow rates can shed light on the initial reaction of blood to foreign materials. Miniaturized extracorporeal circuit devices with heparin-coated gas (PMP) or heat-exchange (PET) hollow-fiber membranes were exposed to high and low flow rates. Hemocompatibility and adsorption of plasma proteins were measured after one minute to six hours using mass spectroscopy analyses. Approximately 150–200 different proteins were present on the membranes, with almost no variation in the 10 most abundant proteins. Protein adsorption to the membrane types did not vary to a large extent, but a decreased flow rate significantly reduced the differences in protein adsorption between both membrane types and led to the adhesion of significantly higher amounts of inhibitory proteins C1INH and α1-AT. At the higher flow rate, coagulation-associated proteins adsorbed significantly more to PET membranes, whereas complement-activating-related proteins adsorbed more on PMP membranes. Our results highlight the importance of analyzing all circuit components to understand the activation of blood components during ECMO. The primary contributor to increased protein adsorption and activation of blood components was an increased flow rate. Therefore, flow rate adjustments should ideally aim to achieve optimal oxygenation levels of around 80% while minimizing protein adsorption and blood activation during ECMO. Notably, at a low flow rate, PMP HFM exhibited a significant increase in binding of complement and inflammation inhibitors, suggesting a potential benefit of lowering the flow rate apart from the general reduction in protein adsorption.

## 1 Introduction

Extracorporeal membrane oxygenation (ECMO) is a potentially life-saving procedure used to support the lungs of critically ill patients. With its tubes and cannulas, pump, heat exchanger, and hollow fiber membrane (HFM) oxygenator, ECMO creates a large artificial surface that comes into contact with blood. Of all components, the oxygenator accounts for the largest part (>90%) of the blood-contacting area with 0.8–2.5 m^2^. In addition, the tubes form a surface area of 0.05–0.15 m^2^ (1). Due to this large foreign surface area and the necessary systemic anticoagulation, hemostatic changes often occur during ECMO, including both bleeding and thrombotic events (2).

To prevent complications during ECMO and to increase hemocompatibility, the ECMO systems are coated. Heparin-albumin coatings are mainly used in long-term applications for up to 30 days (3). For this purpose, tip-to-tip coating is usually used, which means that all blood-contacting surfaces of the ECMO are modified (4). When developing new coatings and testing hemocompatibility, great attention is usually paid to HFMs for gas exchange. In contrast, the membranes for heat exchange receive little attention, but they are also a significant part of the blood-contacting surface. In addition to the different materials that come into contact with blood during ECMO treatment, the selected flow rate has also an influence on hemocompatibility (5). In adults, the flow rate during ECMO is 4–6 L/min (60–80 ml/kg/min) (6). This flow rate is necessary to maintain adequate gas exchange. During weaning, the flow rate is gradually reduced to less than 30%. These recommended flow rates are well documented (7); however, it is not well understood how different flow rates affect coagulation and hemolysis.

The flow rate can determine the speed of cell and protein adsorption on the surface. Furthermore, by varying the flow rate, the degree of receptor expression on platelets and leukocytes can change (8). Since platelets play a crucial role in thrombus formation, numerous studies have focussed on the impact of flow rate on platelet behavior (9–14). High flow rates and the associated increase in shear forces led to increased deposition of platelets (15, 16). Low flow rates had the opposite effect (17). On the other hand, it has been described that fibrin deposition decreased with increasing wall shear rate (18).

The importance of flow is widely known, but the current understanding of the influence of flow is mainly limited to platelets. Less is known about the effects of flow rate on the activation of the coagulation system. However, it is known that the formation of factor Xa increases with increasing shear rate (19) and that thrombin formation is influenced by flow velocity (20). In addition, cannulas and centrifugal pumps required for ECMO support can alter fluid dynamics by creating turbulent flows and high shear stresses, thus altering hemodynamic effects (21, 22). This results in damage to circulating blood cells and hemostatic proteins. In addition, it has been shown that extracorporeal life support can lead to acquired von Willebrand syndrome, which is characterized by the loss of high molecular weight (HMW) multimers of von Willebrand factor (vWF) due to high shear stress and leads to impaired binding of vWF to collagen and platelets (23). This leads to impaired primary hemostasis and patients develop a bleeding tendency.

This study aims to assess the material- and flow rate-dependent plasma protein binding over time, and to investigate the effects of plasma protein binding on thrombosis and inflammation by detecting various hemocompatibility markers. For this purpose, a mock-loop system was developed in which polymethyl pentene (PMP) gas exchange HFM membranes and polyethylene terephthalate (PET) heat exchange membranes were incubated with human whole blood or human plasma at two different flow rates (0.2 L/min and 1 L/min) for six hours.

## 2 Materials and methods

### 2.1 Miniature devices with heparin-coated hollow-fiber oxygenator membranes

Miniature devices were used with gas exchange or heat exchange membranes. The gas exchange membrane was a PMP hollow-fiber membrane (OXYPLUS™, 3M Membrana, Wuppertal, Germany) and the heat exchange membrane was a PET hollow-fiber membrane (HEXPET™, Capillary, Type 60/670, 3M Membrana, Wuppertal, Germany). The miniature device components were individually coated as previously described (24) and then assembled under sterile conditions. Heparin-coated PMP or PET membrane pieces (14 × 10 cm, approx. 210 cm^2^ surface area) were placed in a 3/8-in (0.95 cm) polyvinylchloride (PVC) tube (Raumedic, Helmbrechts, Germany) with a 3/8-in–1/4-in (0.95–0.65 cm) polycarbonate straight connector at both ends. The miniature devices were covalently coated with the EBS (Ension Bioactive Surface) coating technology as described before (24). The fundamental concepts of the EBS coating are described by Johnson et al. (25).

### 2.2 Blood and plasma sampling

The citrated plasma and heparinized whole blood were obtained from the donor pool of the Transfusion Service of the University Hospital Tübingen. The frozen citrated plasma was mixed with 3 IU/ml sodium heparin (25,000 IU/5 ml; LEO Pharma GmbH, Neu-Isenburg, Germany) after thawing and recalcified with calcium chloride (Baxter, Glenview, USA) depending on the citrate concentration.

For the hemocompatibility studies, 500 ml fresh heparinized (3 IU/ml) whole blood obtained directly from the transfusion service was used. The whole blood was diluted with 100 ml diluent solution containing 1.3% glucose solution (Delta-Pharma GmbH, Pfullingen, Germany) and 53 mM NaHCO_3_ (Braun Melsungen AG, Melsungen, Germany) in ringer lactate solution (Fresenius, Bad Homburg, Germany).

### 2.3 Experimental setup

To characterize the adsorption of plasma proteins and the hemocompatibility parameters on heparin-coated PMP and PET membranes at different blood flows, a 1/4-in PVC tubing system (Raumedic, Helmbrechts, Germany) was set up with the miniature devices as described previously (24). For time-dependent protein adsorption analyses on heparin-coated PMP and PET membranes, separate circulation systems were run with two different flows (0.2 L/min and 1 L/min). The flow rates were adjusted to the miniature model with a smaller tube diameter to simulate the flow rates of the patient oxygenators. The higher flow corresponds approximately to the normal cardiac output of an adult and thus the normal ECMO pumping rate of approximately 4–6 L/min. The reduced flow of 0.2 L/min corresponds approximately to the ECMO flow during weaning (approximately 1.5 L/min). For this purpose, the system with the miniature device was filled with plasma and stopped after different time points. After the appropriate incubation time, the system was rinsed with 1 L of 0.9% NaCl solution and the washed membrane was removed. The adsorbed proteins were desorbed as previously described (24).

### 2.4 Hemocompatibility tests

For the experiments with whole blood, a blood reservoir was added to the circulation system described earlier (24). To determine the baseline values, a baseline sample was taken before the start of incubation. The diluted whole blood described in Section 2.2 was divided to allow simultaneous determination of the two different membrane types (PMP and PET) with the same donor blood. The experiments were carried out with a blood flow of 1 L/min and 0.2 L/min and a temperature of 37°C. Blood was collected from the system at different time points (1, 5, 10, 30, 60, 90, 180, and 360 min after the start of circulation). Blood cell counts were determined directly using an automated cell counter (ABX Micros 60, Horiba Medical, Kyoto, Japan). Furthermore, collected serum or plasma samples were cryopreserved and later the activation of coagulation, complement system, platelets, and inflammation were determined by ELISAs. All ELISAs were performed according to the manufacturer's instructions and the following ELISAs were used: SC5b-9 (MicroVue™ Complement, Quidel, Osteomedical GmbH, Sissach, Switzerland), thrombin-antithrombin III complex (TAT) (Enzygnost® TAT micro, Siemens Healthcare, Erlangen, Germany), polymorphonuclear (PMN) elastase (PMN elastase ELISA, Demeditec Diagnostics, Kiel, Germany), and β-thromboglobulin (β-TG) (Asserachrome® β-TG, Diagnostica Stago, Parsippany, NJ, USA). In addition, protein adsorption to the membranes was determined at the end of whole blood incubation as described in Section 2.3.

### 2.5 Mass spectrometry (MS) analysis

SDS PAGE short gel purification was run and in-gel digestion with trypsin was conducted as described previously (26). Extracted peptides were desalted using C18 StageTips (27) and subjected to LC-MS/MS analysis. LC-MS/MS analyses were performed on an Easy-nLC 1200 UHPLC (ultra-high performance liquid chromatography) (Thermo Fisher Scientific) coupled to an QExactive HF Orbitrap mass spectrometer (Thermo Fisher Scientific) as described elsewhere (28). Peptides were eluted with a 60 min segmented gradient at a flow rate of 200 nl/min, selecting the 20 most intensive peaks for fragmentation with HCD (higher-energy collisional dissociation). The MS data was processed with MaxQuant software suite v. 1.5.2.8 and v.1.6.7.0 (29) The LFQ (label-free-quantification) was used for analyses. Database search was provided against human (96,817 entries) UniProt database using the Andromeda search engine (30).

### 2.6 Statistics

Experiments were performed with the blood from four independent donors (*n* = 4) and three different plasma (*n* = 3 independent experiments, each plasma consisted of a pool from 3 donors). Significant differences were analyzed using unpaired two-tailed Student's *t*-tests assuming equal variance. Statistical significance was defined as *p* < 0.05 and statistical analysis was performed using Microsoft Excel 365 (Microsoft, Albuquerque, USA) and GraphPad Prism version 6.01 (GraphPad Software Inc., La Jolla, CA, USA).

## 3 Results

In this research work, the influence of two different membranes, PMP gas exchange or PET heat exchange membranes, and different flow rates (1.0 L/min and 0.2 L/min) on the surface plasma protein binding over time was investigated. Only heparin-coated miniature devices were used. Part of the data, namely heparinized PMP devices with 1.0 L/min, were discussed previously (24), but all experiments shown here were performed at the same time. To be able to draw conclusions about the effects of various endogenous defense reactions, various markers of blood coagulation, the complement system, inflammation, and platelet activation were determined using ELISAs in addition to the detection of adsorbed proteins using MS.

### 3.1 Comparison of total numbers of desorbed proteins

In total, 532 different desorbed proteins were detected by MS in the four conditions. A full list of desorbed proteins measured by MS can be found in Supplementary Tables S1–S4. The total number of different proteins found at both membrane types and at the low and high flow rates are listed in Table 1. The highest total number of different proteins was detected for PET (heat exchange) membranes at 1 L/min while both membranes (PMP and PET) at lower flow rates (0.2 L/min) showed similar protein numbers. However, the number of proteins present at all individual time points showed a different result. Here, the lower flow rates showed higher proportions of proteins that were present at each time point at both membrane types that at the higher flow rates.

**Table 1:** Number of desorbed proteins after contact with plasma that were present at all time points on heparin-coated PMP and PET membranes at different flow rates (1 L/min and 0.2 L/min) presented as percentage of the total protein number found in general and present at the respective condition.

Type of membrane	Flow rate	# of different proteins	% of proteins present at all time points	
			Per condition	From total
PMP	1 L/min	373	40.8	29.0
	0.2 L/min	311	47.9	28.4
PET	1 L/min	403	35.2	27.0
	0.2 L/min	306	48.7	28.4

**Figure 1:**
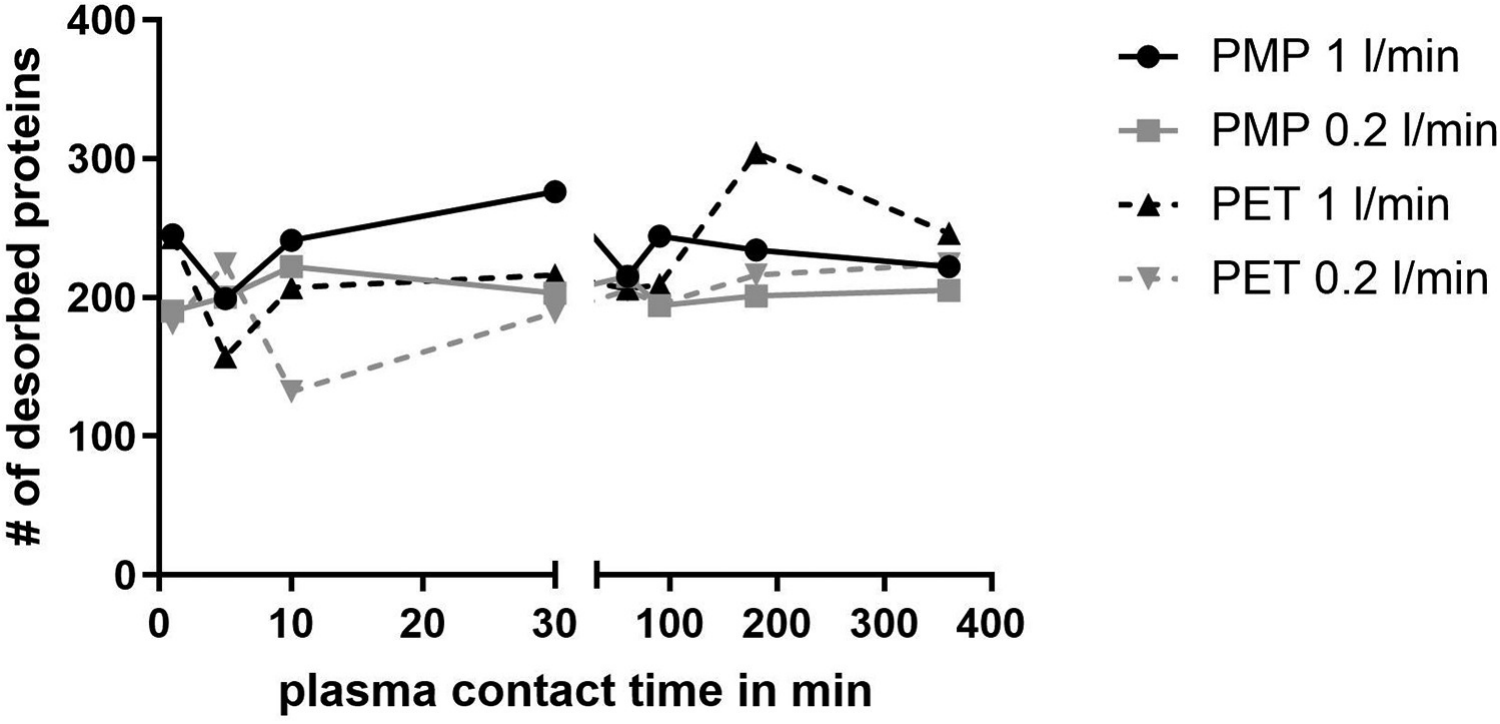
Number of different desorbed proteins after plasma contact over time on different surfaces (PMP and PET) and under different conditions (flow rates 1 L/min and 0.2 L/min).

**Figure 2:**
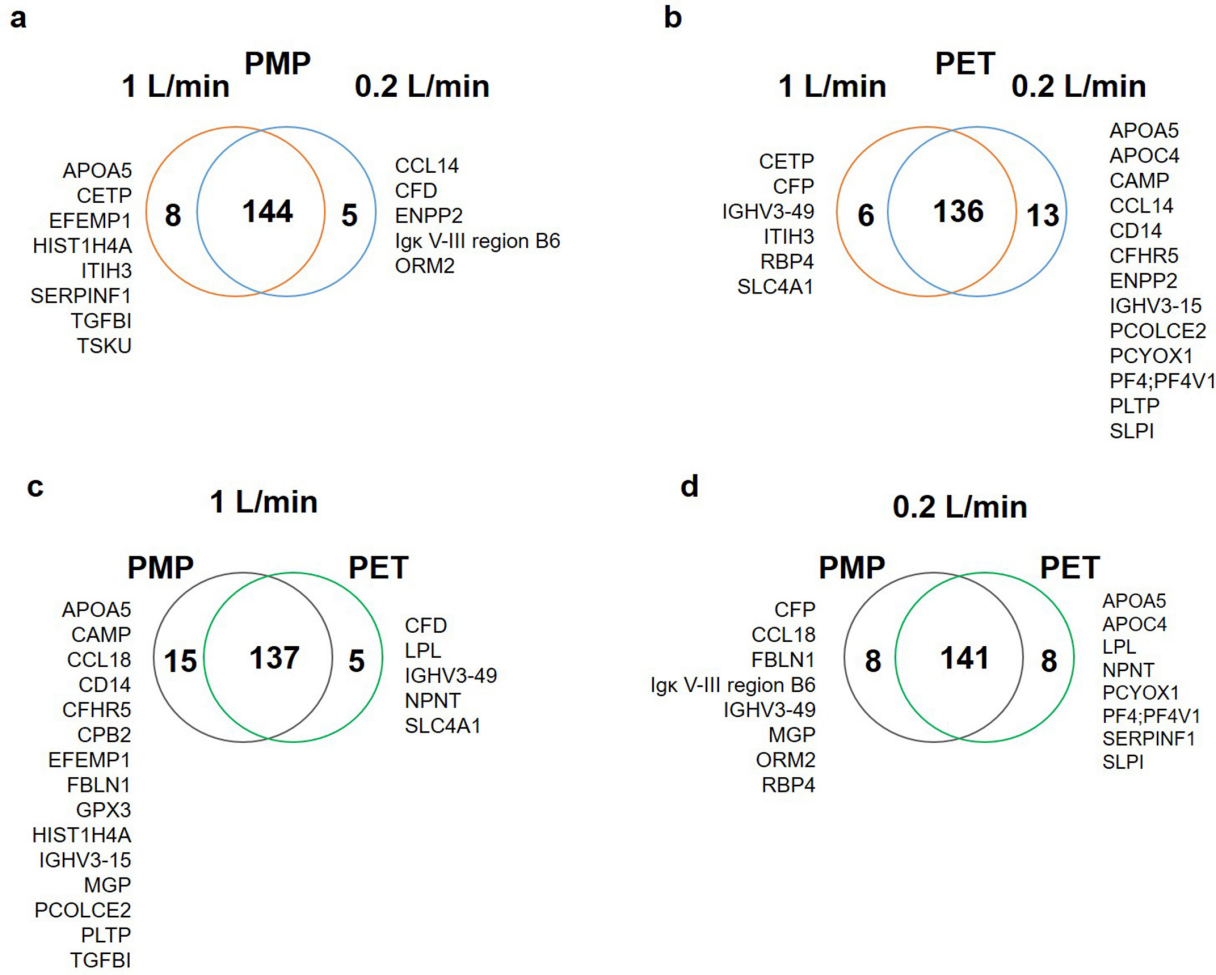
Venn-Diagrams presenting the number of proteins present at all time points in the individual conditions in comparison. **(a)** On the PMP membrane 144 proteins were present at both flow rates, whereas 8 proteins were only seen at all time points at high flow, and 5 at low flow. **(b)** On the PET membranes 136 proteins were present at all time points, but 6 only at the higher flow and 13 only at the lower flow rate at all time points. **(c)** At the high flow rate 137 proteins were found at each time point on both membrane types, but 15 were present only on PMP at each time point, and 5 on PET at each time point. **(d)** At the lower flow rate 141 proteins were found at each time point on both membrane types, but 8 were not present at all time points on PMP or PET, respectively.

The numbers of proteins at each time point are shown in Figure 1. Here, the PMP membranes at the high flow rate had mostly the highest number of proteins desorbed. Already here it becomes apparent that the influence of different membrane materials is not high, if both are heparin-coated, especially at a low flow rate. While at a faster flow rate of 1 L/min, there was occasionally a higher difference in the number of bound proteins (1 L/min, black lines), the number of bound proteins at a flow rate of 0.2 L/min was almost identical (0.2 L/min, grey lines). Comparing the different flow rates, it is evident that a faster flow rate also leads to a higher number of bound proteins on the PMP membrane; this trend was evident throughout the entire time. A similar behavior was observed with the PET membrane. However, after 5 min the opposite behaviour was observed once, with a lower number of proteins desorbed from the high-flow device. In the period between 10 and 90 min, the protein number was almost identical before it increased at 180 min with a higher flow rate and then settled again at a similar level after 360 min. Overall, 133 proteins were found at each individual time point in all conditions. The number of proteins differing between conditions can be seen in the Venn-Diagrams (Figures 2a–d). It is apparent that only few (5–15) differed at most in their binding profile. For each parameter comparison, i.e., flow rates on PMP (Figure 2a) or PMP (Figure 2b), and materials at 1 L/min (Figure 2c) or 0.2 L/min (Figure 2d), that are consistently present at all time points in one condition but absent at some time points in the other are named.

### 3.2 Identifying the most abundant proteins on different membrane types and flow rates

To determine the composition of the protein layer on the membranes, the relative amount of each protein in relation to the other proteins was determined. This can provide information about the statistically most likely present proteins and binding partners, and thus define the influence of the material and the flow rate more accurately. Tables 2–5 show the ten most abundant proteins on the PMP and PET membranes at flow rates of 1 L/min and 0.2 L/min, respectively. The order of abundance of all proteins is shown in Supplementary Tables S1–S4.

**Table 2:** The ten most abundant proteins on heparin-coated PMP membranes incubated with a flow rate of 1 L/min.

PMP 1 L/min	1 min	5 min	10 min	30 min	60 min	90 min	180 min	360 min
1	APOB	ALB	APOB	ALB	FGA	APOB	ALB	FGA
2	ALB	FGG	FGG	APOB	APOB	FGA	FGG	FGB
3	SERPINC1	FGA	ALB	FGG	FGB	FGB	APOB	APOB
4	FGG	FGB	FGA	FGA	FGG	FGG	FGA	FGG
5	FGA	APOB	SERPINC1	FGB	ALB	ALB	FGB	ALB
6	APOE	FN1	FGB	SERPINC1	SERPINC1	SERPINC1	ITIH4	ITIH4
7	FGB	SERPINC1	APOE	APOE	FN1	APOE	C3	FN1
8	LBP	C3	FN1	C3	APOE	C3	FN1	C3
9	FCN2	APOE	FCN2	TF	C3	FN1	SERPINC1	SERPINC1
10	FN1	TF	LBP	FN1	ITIH4	ITIH4	APOE	APOE

Reproduced with permission from “The ten most abundant proteins on heparin-coated PMP membranes” by Katharina Große-Berkenbusch, Meltem Avci-Adali, Madeleine Arnold, Linda Cahalan, Patrick Cahalan, Ana Velic, Boris Maček, Christian Schlensak, Hans Peter Wendel and Sandra Stoppelkamp.

**Table 3:** The ten most abundant proteins on heparin-coated PMP membranes incubated with a flow rate of 0.2 L/min.

PMP 0.2 L/min	1 min	5 min	10 min	30 min	60 min	90 min	180 min	360 min
1	ALB	ALB	ALB	FGA	FGA	ALB	ALB	APOB
2	FGA	FGB	APOB	FGB	FGB	FGA	APOB	FGA
3	FGB	FGA	FGA	APOB	FGG	FGB	FGA	FGB
4	APOB	FGG	FGB	FGG	APOB	FGG	FGB	FGG
5	FGG	FN1	FGG	ALB	ALB	APOB	FGG	ALB
6	SERPINC1	APOB	SERPINC1	SERPINC1	SERPINC1	C3	SERPINC1	APOE
7	FN1	SERPINC1	FN1	APOE	FN1	FN1	APOE	FN1
8	APOE	APOE	APOE	FN1	APOE	SERPINC1	C3	SERPINC1
9	LBP	C3	FCN2	FCN2	FCN2	APOE	FCN2	C3
10	C3	IGHG1	ANG	C3	C3	FCN2	FN1	LPA

**Table 4:** The ten most abundant proteins on heparin-coated PET membranes incubated with a flow rate of 1 L/min.

PET 1 L/min	1 min	5 min	10 min	30 min	60 min	90 min	180 min	360 min
1	FGA	FGA	FGA	FGA	FGA	FGA	FGA	FGA
2	FGB	FGB	ALB	FGB	FGB	FGB	FGB	APOB
3	FGG	FGG	FGB	ALB	ALB	ALB	ALB	FGB
4	ALB	ALB	FGG	FGG	FGG	FGG	FGG	FGG
5	APOB	FN1	APOB	APOB	APOB	APOB	APOB	ALB
6	SERPINC1	APOB	SERPINC1	FN1	SERPINC1	C3	C3	ITIH4
7	FN1	SERPINC1	FN1	APOE	FN1	FN1	FN1	FN1
8	APOE	APOE	APOE	SERPINC1	C3	ITIH4	ITIH4	C3
9	LBP	C3	C3	C3	ITIH4	SERPINC1	SERPINC1	SERPINC1
10	C3	ANG	LBP	ITIH4	APOE	APOE	APOE	APOE

**Table 5:** The ten most abundant proteins on heparin-coated PET membranes incubated with a flow rate of 0.2 L/min.

PET 0.2 L/min	1 min	5 min	10 min	30 min	60 min	90 min	180 min	360 min
1	FGA	ALB	FGB	FGA	ALB	FGA	APOB	ALB
2	FGB	FGA	FGA	FGB	FGA	FGB	ALB	APOB
3	FGG	FGB	FGG	FGG	FGB	FGG	FGA	FGA
4	ALB	FGG	ALB	ALB	FGG	APOB	FGB	FGB
5	FN1	APOB	FN1	APOB	APOB	ALB	FGG	FGG
6	APOB	SERPINC1	APOB	SERPINC1	SERPINC1	SERPINC1	SERPINC1	C3
7	SERPINC1	FN1	SERPINC1	APOE	C3	APOE	C3	APOE
8	APOE	APOE	APOE	C3	APOE	FN1	APOE	SERPINC1
9	C3	C3	ANG	LBP	FN1	C3	FN1	FN1
10	TF	TF	C3	FN1	TF	LBP	LBP	TF

The proteins that bind with the highest abundance were very similar for both membrane types and flow rates. Eight (APOB, ALB, SERPINC1, FGG, FGA, FGB, APOE, FN1) out of ten were among the ten most abundant proteins in all conditions (i.e., on both membrane types, flow rates, and at all time points), albeit in a slightly different order. While the order of the most abundant proteins on the PMP membranes (Table 3) and the PET membranes (Table 5) changed frequently over time with the lower flow rate, it was remarkably stable on the PET membrane at a flow rate of 1 L/min (Table 4). FGA was the most frequently bound, followed by FGB, which was the second most bound protein except for 10 min and 360 min. Only minor differences were observed in the order of the subsequent proteins of Table 4.

When comparing the abundance of proteins related to the complement pathways, especially the complement activating proteins C3, C1, ficolins, and MASP1/2, but also the regulatory proteins CFH, vitronectin, and clusterin were found among the ∼50 most common proteins in all samples. Thrombosis-activating proteins that were present in larger abundance in all conditions included fibrinogen, prothrombin (F2), tissue factor (F2), F11, kininogen, and vWF, as well as the inhibitory proteins ATIII, protein C inhibitor (PCI), and α-2-macroglobulin (A2M) and the fibrinolysis-related proteins fibronectin and inter-alpha-trypsin inhibitor proteins (ITIH2/3/4).

### 3.3 Differences in protein binding over time

**Figure 3:**
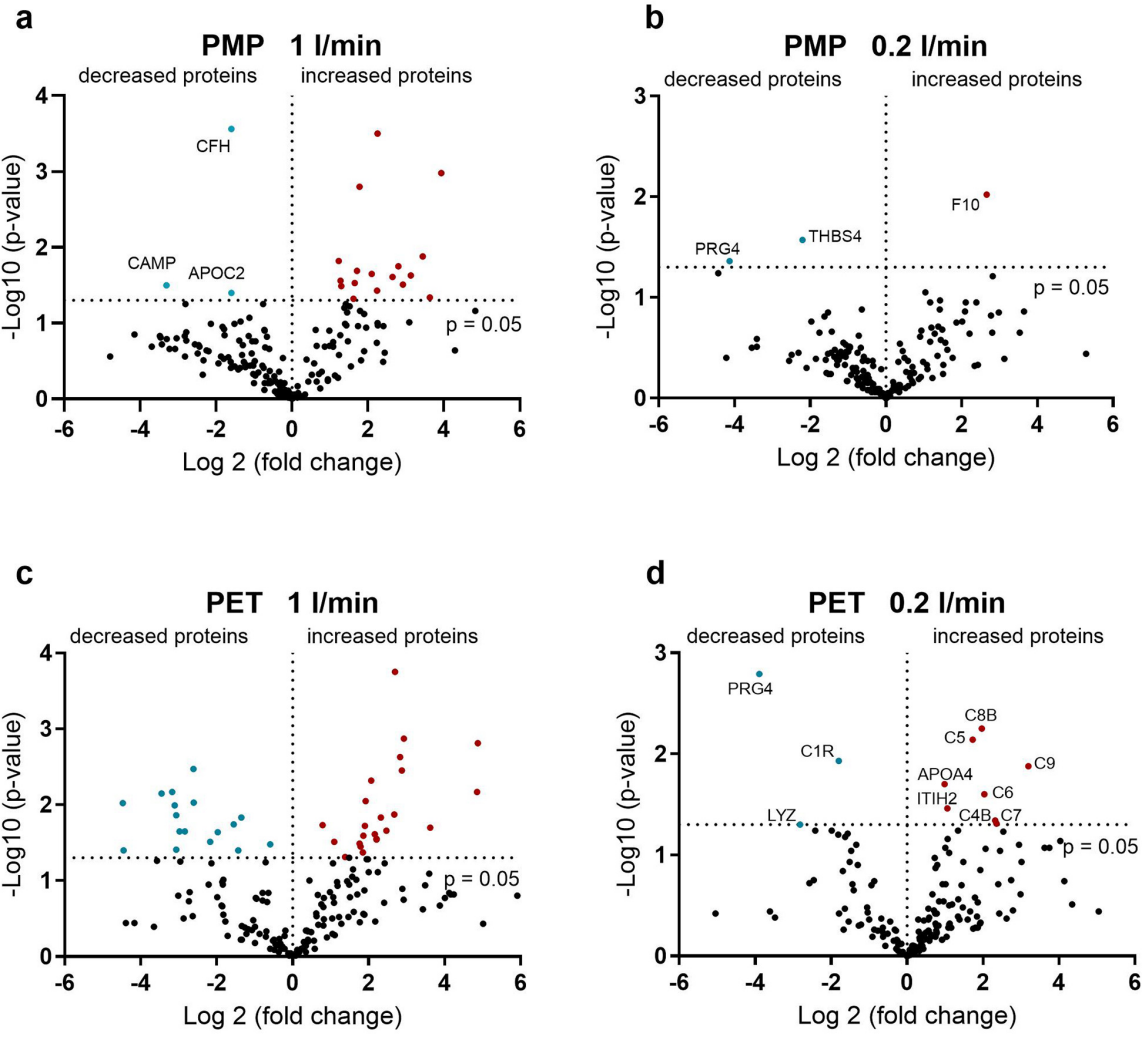
Volcano plot showing the change in protein abundance after 360 min compared to one minute. **(a)** On the PMP membranes at a high flow rate, some of the proteins showed an increasing abundance over time. **(b)** On the PMP membranes at a low flow rate, almost all proteins showed a constant binding profile over time. **(c)** On the PET membranes at a high flow rate, both an increase and a decrease in protein binding over time were observed. **(e)** On the PET membrane at a low flow rate, a lower number of proteins were detected with an increase and a decrease over time. Significant differences were analyzed using unpaired two-tailed Student's *t*-tests assuming equal variance (significance level *p* < 0.05), *n* = 3.

Although the most frequently bound proteins were relatively stable over time for both membrane types and at both flow rates (Tables 2–5), clear differences were observed when comparing the relative abundance (LFQ) of proteins present at all time points over time. The significant changes occurring between 1 and 360 min indicate the remodeling of the protein layer (Figure 3). A stronger remodeling of the protein layer occurred with higher flow rates on both membrane types (Figures 3a vs. b,c vs. d). An interesting observation here was the higher level of significant changes in protein binding over time on the PET membranes compared to the PMP membranes at the same flow rates (Figures 3a vs. c,b vs. d). While only three proteins (CFH, CAMP, and APOC2) with a significantly decreased (3-fold lower) binding abundance over time were detected on the PMP membrane at a high flow rate (Figure 3a), significantly decreased binding abundance over time occurred more frequently and with larger (up to 20-fold) changes on the PET membrane (Figure 3c). Increased protein abundance on the PMP membrane was mostly observed for lipid transport related proteins (CETP, PLTP), inhibitors of trypsin (ITIH3 and 4) and chymotrypsin (SERPIND1), fibrinogen, von Willebrand factor, and complement inhibitors clusterin and vitronectin as well as complement factor 9 (C9).

By reducing the flow rate, significant changes in binding frequency over time can be almost eliminated for both membrane types. However, at low blood flow, the few proteins that increase significantly over time are of crucial importance. On the PMP membrane, binding of the central factor of the coagulation cascade, FX, increased 6-fold over time. In contrast, on the PET membrane with lower flow rate, proteins of the complement system mainly increased significantly over time, with C9 increasing the most (9-fold).

### 3.4 Identifying the effect of flow rate on plasma protein binding

**Figure 4:**
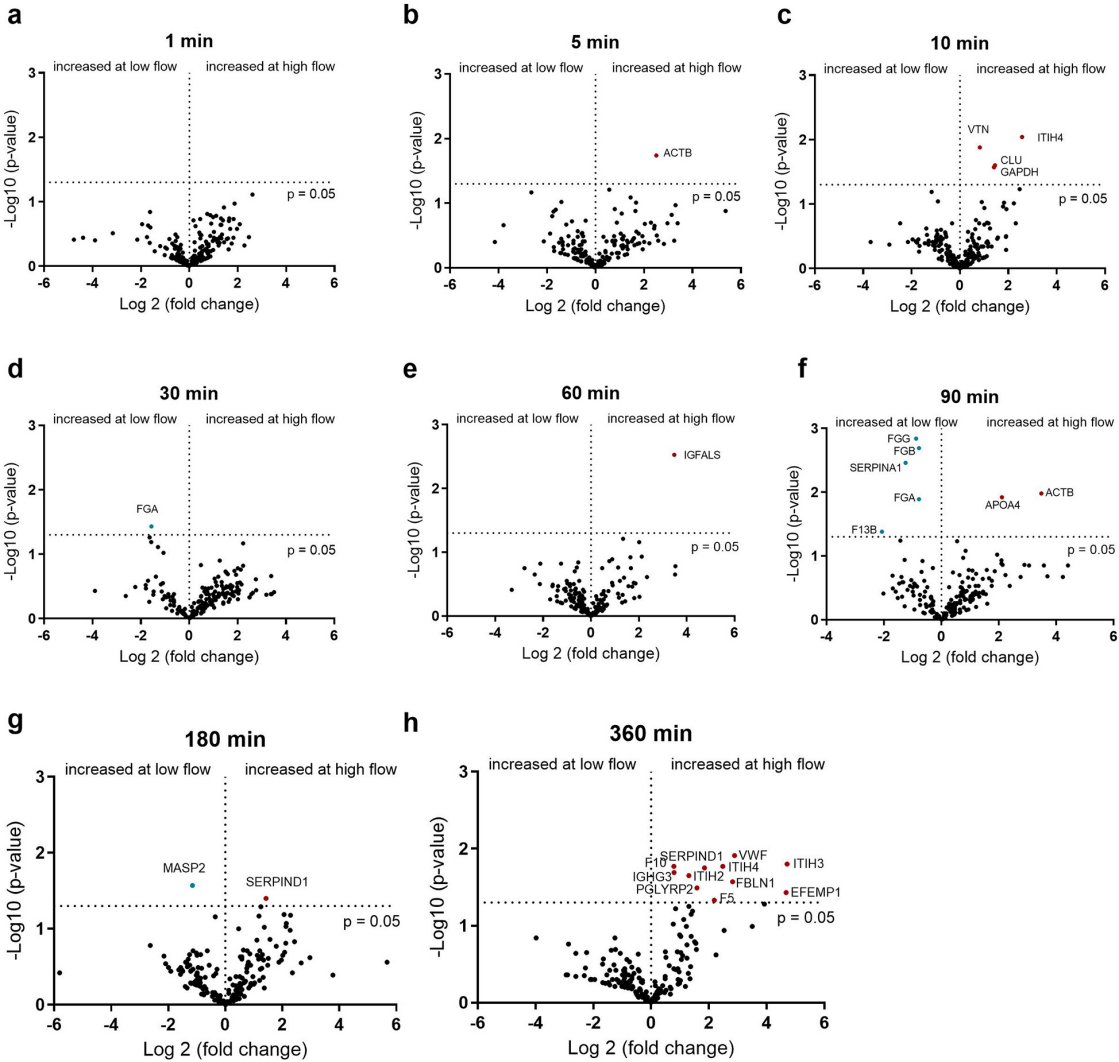
Volcano plot showing the changes in protein abundance with slow (0.2 L/min) vs. high flow (1 L/min) rate on heparin-coated PMP membrane at 1 **(a)**, 5 **(b)**, 10 **(c)**, 30 **(d)**, 60 **(e)**, 90 **(f)**, 180 **(g)** and 360 **(h)** minutes. Significant differences were analyzed using unpaired two-tailed Student's *t*-tests assuming equal variance (significance level *p* < 0.05), *n* = 3. Proteins marked in red bind significantly more at higher flow rate and proteins marked in blue bind significantly more at lower flow rate.

Since different degrees of protein remodeling were observed during the 6 h under the four conditions, the individual time points were investigated in more detail. First, the adsorbed proteins were compared between a slow (0.2 L/min) and fast (1 L/min) flow rate with PMP and PET membranes. The effects of flow rate on protein adsorption on the PMP membrane are shown in Figure 4. At most time points, no significant increase in protein adsorption was observed at low flow rates. Significantly increased protein binding was detected for some proteins [α-1-antitrypsin (SERPINA1), FXIII (F13B), and fibrinogen] after 90 min at a low flow rate on PMP membranes (Figure 4f). At a low flow rate, increased fibrinogen binding was also observed after 30 min (Figure 4d), as well as increased MASP1 binding after 180 min (Figure 4g). In particular, after 360 min at a faster flow rate (Figure 4h), increased numbers of significantly elevated proteins, including proteins of the coagulation cascade (VWF, F10, SERPIND1, F5) were detected.

**Figure 5:**
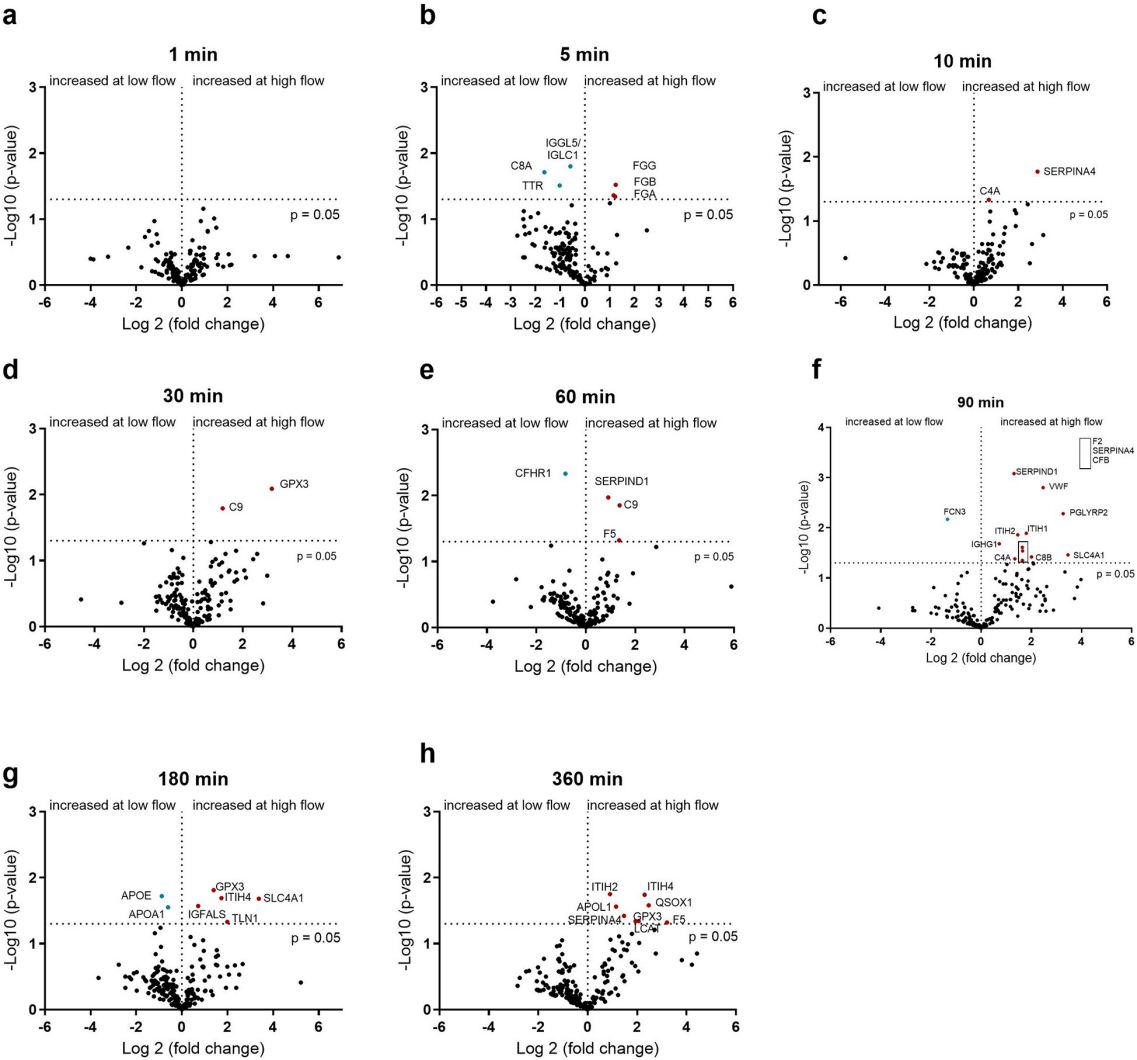
Volcano plot showing the changes in protein abundance with slow (0.2 L/min) vs. high flow (1 L/min) rate on heparin-coated PET membrane at 1 **(a)**, 5 **(b)**, 10 **(c)**, 30 **(d)**, 60 **(e)**, 90 **(f)**, 180 **(g)** and 360 **(h)** minutes. Significant differences were analyzed using unpaired two-tailed Student's *t*-tests assuming equal variance (significance level *p* < 0.05), *n* = 3. Proteins marked in red bind significantly more at higher flow rate and proteins marked in blue bind significantly more at lower flow rate.

The influence of the flow rate on the PET membrane is shown in Figure 5. In contrast to the PMP membrane, differences in protein adsorption can already be seen after 5 min. Here, fibrinogen binds significantly more at a higher flow rate (Figure 5b). This is in contrast to the PMP membrane, where fibrinogen was bound significantly more at a lower flow rate. Within the first 60 min, the 5 min time point showed the most alterations in protein abundance due to flow intensity and an increased C9 binding after 30 and 60 min at a higher flow rate could be detected. From 90 min onwards, several proteins again showed increased binding due to a faster flow rate. These included proteins of the coagulation cascade (VWF, F2) and complement factor B (CFB).

### 3.5 Identifying the effect of the surface on plasma protein binding

**Figure 6:**
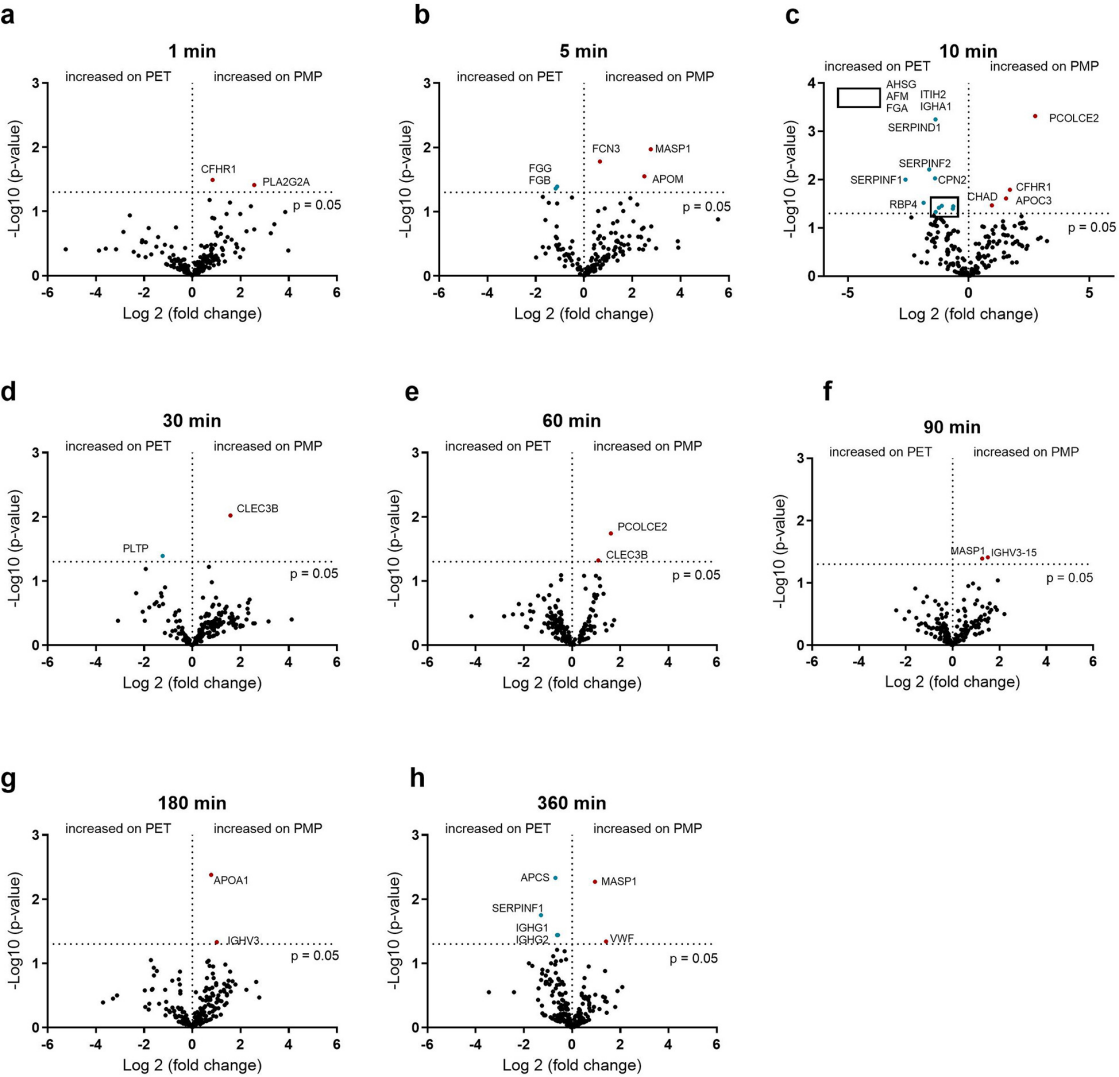
Volcano plot showing the changes in protein abundance on heparin-coated PMP membrane vs. heparin-coated PET membrane with a high flow from 1 L/min at 1 **(a)**, 5 **(b)**, 10 **(c)**, 30 **(d)**, 60 **(e)**, 90 **(f)**, 180 **(g)** and 360 **(h)** minutes. Significant differences were analyzed using unpaired two-tailed Student's *t*-tests assuming equal variance (significance level *p* < 0.05), *n* = 3. Proteins marked in red bind significantly more on PMP membrane, proteins marked in blue bind significantly more on PET membrane. The 5 proteins in the box in **(c)** are named in the upper left quadrant of the diagram due to space issues.

In addition to the influence of the flow rate, the dependence of protein binding on the membrane type was also investigated. The differences in protein binding between PMP and PET membranes at a fast flow rate are shown in Figure 6. In the initial phase, after 5 min, FCN3, MASP1, and APOM were significantly higher on PMP membranes, and significantly higher fibrinogen binding was detected on PET membranes. In general, the greatest differences in binding frequency were observed after 10 min, indicating the strongest influence of the material at this time. Thereafter, only a few differences (mainly on the PMP membrane) were observed. This shows that more significant protein changes over the entire time were seen on PMP membranes at the high flow rate, but a stronger remodeling occurred on PET membranes at 10 min.

**Figure 7:**
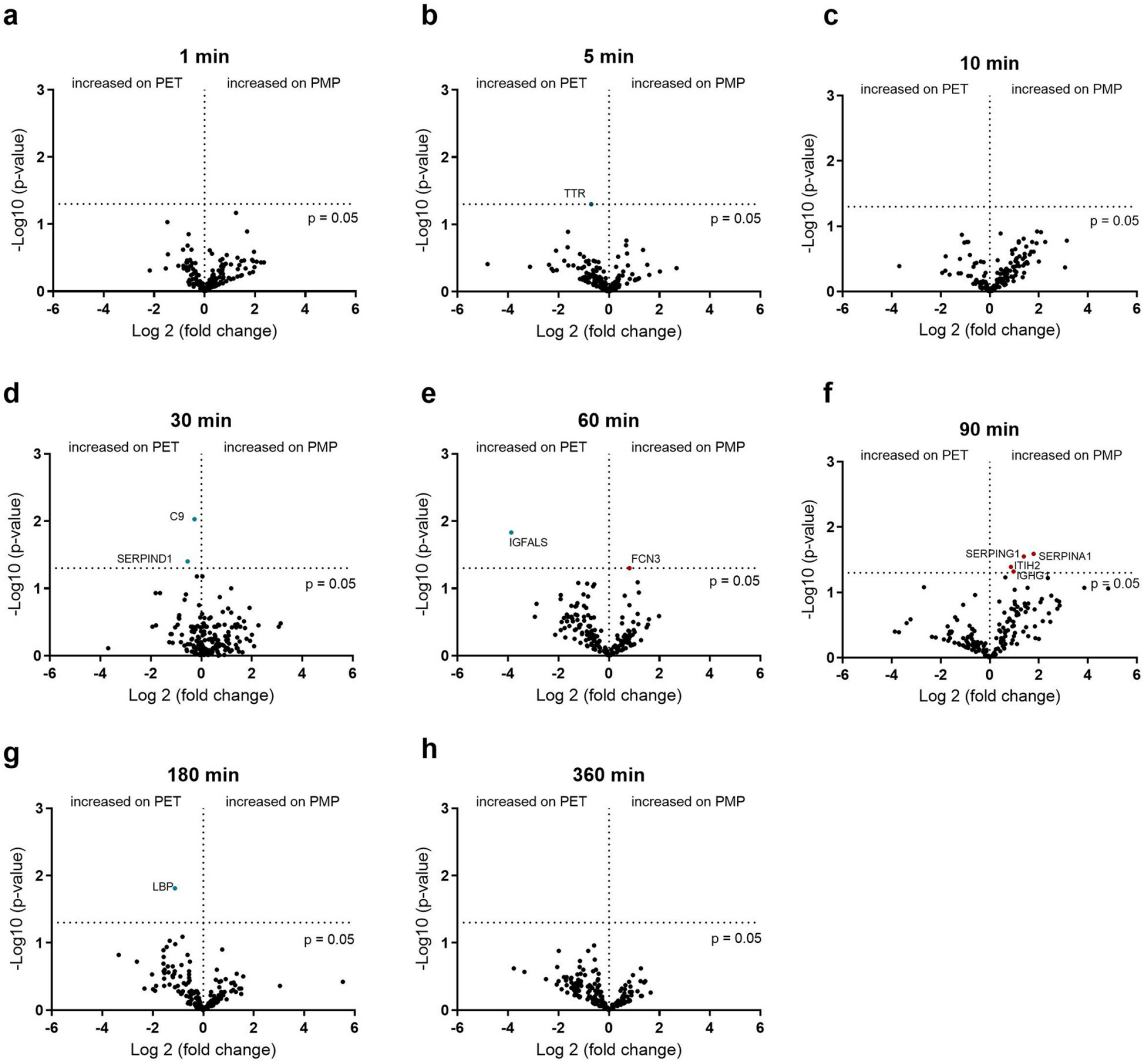
Volcano plot showing the changes in protein abundance on heparin-coated PMP membrane vs. a heparin-coated PET membrane with a low flow from 0.2 L/min at 1 **(a)**, 5 **(b)**, 10 **(c)**, 30 **(d)**, 60 **(e)**, 90 **(f)**, 180 **(g)** and 360 **(h)** minutes. Significant differences were analyzed using unpaired two-tailed Student's *t*-tests assuming equal variance (significance level *p* < 0.05), *n* = 3. Proteins marked in red bind significantly more on PMP membrane, proteins marked in blue bind significantly more on PET membrane.

If the flow was reduced to 0.2 L/min, the membrane type played almost no role, in contrast to a higher flow rate of 1 L/min. The small differences in the binding abundance are shown in Figure 7. Although only very few proteins differed significantly in their binding abundance, these could be of significance. The increased binding of C9 and heparin co-factor 2 (SERPIND1) on the PET membrane after 30 min, the increased binding of ficolin-3 (FCN3) after 60 min as well as the increased binding of C1-esterase inhibitor (SERPING1) and α-1-antitrypsin (SERPINA1) on the PMP membrane after 90 min should be emphasized here.

The individual binding profiles of all coagulation and complement components that showed significant differences in Figures 3–6 are shown in Supplementary Figures S1 (complement) and S2 (coagulation).

### 3.6 Comparison of the hemocompatibility

**Figure 8:**
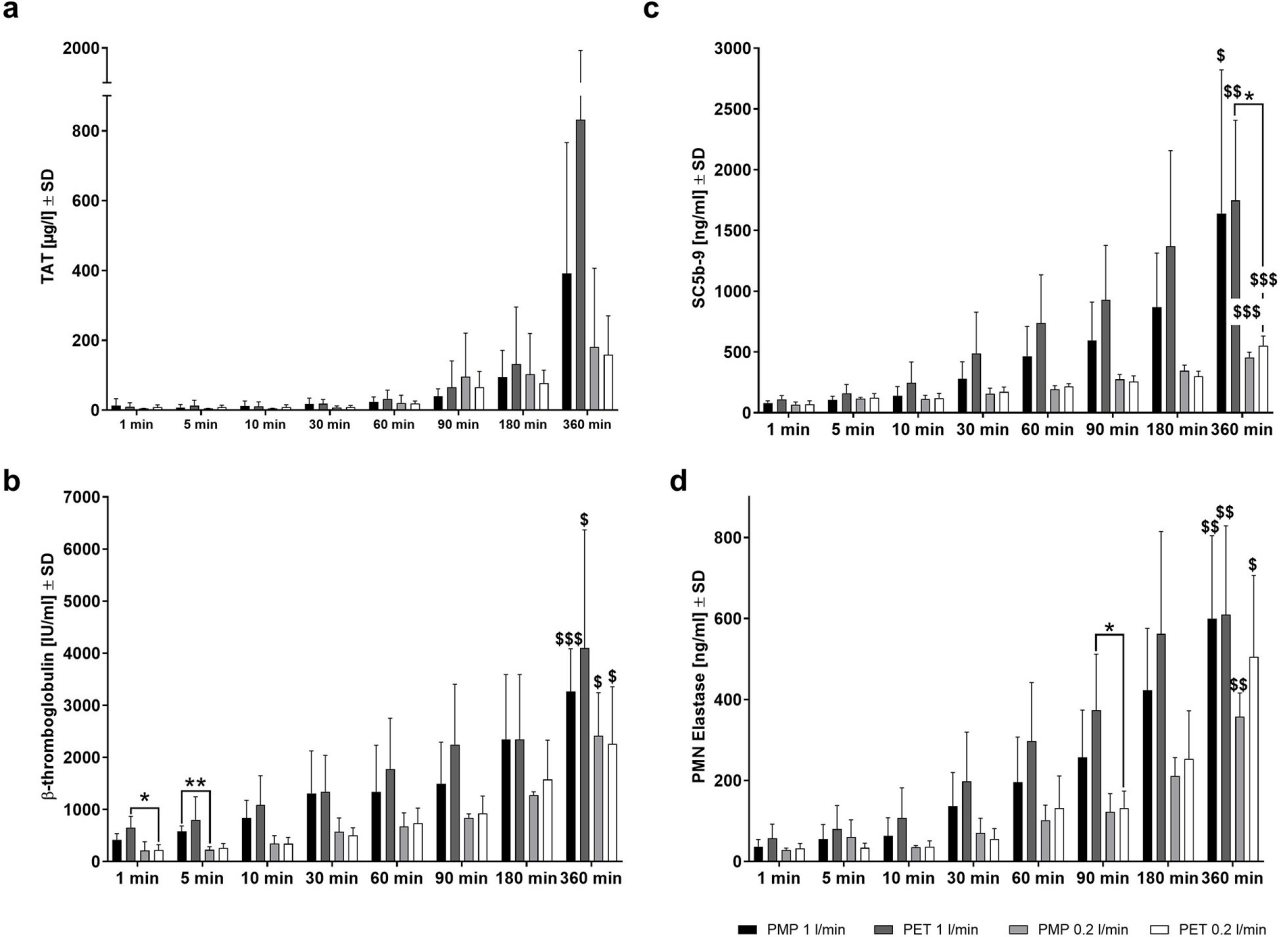
Hemocompatibility parameters measured after whole blood incubation at different time points. Plasma levels of **(a)** coagulation activation (TAT), **(b)** inflammation activation (PMN-elastase), **(c)** complement activation (SC5b-9), and **(d)** platelet activation (β-TG) were measured in a mock loop system with either a heparin-coated PMP membrane or a heparin-coated PET membrane and at a high (1.0 L/min) or low (0.2 L/min) flow rate. Differences were detected by unpaired two-tailed Student's *t*-test *p < 0.05, **p < 0.01 show significant differences between materials or settings within the same time point; “$” denotes significant differences compared to 1 min of the same material or setting, $ *p* < 0.05, $$ *p* < 0.01, $$$ *p* < 0.001, *n* = 4.

To determine the effects of protein binding on the course of the body's various defense reactions, the blood cell counts and plasma levels of various hemocompatibility markers were determined over time (Figure 8). Blood cell counts and hematological parameters were assessed at all time points. The numbers of white blood cells, red blood cells, platelets, as well as hemoglobin and hematocrit levels remained constant throughout the experiment, with no significant differences over time or between high and low flow rate time points and the different membrane types (data not shown). Inflammation (PMN elastase), complement (SC5b-9), coagulation (TAT complex), and platelet (β-TG) activation markers increased over time with both membrane types and flow rates, but only after 6 h a significantly increased activation of inflammation, complement system, and platelets were detected (*p* < 0.05 labeled with $). Although not statistically significant, the highest TAT levels were also observed after 6 h of incubation.

A lower flow rate with PMP and PET membranes resulted in lower PMN elastase, SC5b-9, and β-TG levels across the different time points than at higher flow rate. At 360 min, TAT values were also lower in samples obtained from low flow rate conditions for both membrane types. The incubation of PET membranes with the lower flow rate led to significantly lower platelet activation (β-TG) at 1 min (*p < 0.05) and inflammatory activation (PMN elastase) at 90 min (*p < 0.05) (Figure 8d). Furthermore, at 5 min, significantly lower β-TG levels were observed in samples incubated with PMP membranes at a low flow rate compared to a high flow rate (**p < 0.01) (Figure 8b). This is remarkable as no significant differences in protein binding due to flow rates were observed at this early stage (Figure 4). Complement activation was significantly reduced in blood samples incubated with the PET membrane after 360 min at a lower flow rate (*p < 0.05) (Figure 8c). This was also indicated by significantly increased C9 binding to the PET membrane after 30 (Figure 5d) and 60 min (Figure 5e) of plasma contact at higher flow rates and increased C8B binding after 90 min (Figure 5f).

## 4 Discussion

In this study, the influence of different HFM membrane materials and flow rates on the binding of plasma proteins over time was investigated for the first time. Therefore, heparin-coated miniature devices with HFM membranes were used, and the desorbed plasma proteins on HFMs were compared over time using MS. In addition, various markers of blood activation, i.e., coagulation, complement system, inflammation, and platelets were measured using ELISAs. In the miniature devices, heparin was covalently coupled to the surface by a reductive amination reaction between the terminal aldehydes of the heparin chains and the primary amines in the primer matrix. This form of binding is referred to as end-point attachment (EPA) and reflects the simple covalent binding of the heparin molecules to the substrate. EPA results in the specific binding of proteins with a heparin-binding side, as discussed in detail in our previous study (24).

Considering the overall quantity (LFQ) of proteins, a comparable amount of proteins adsorbed to both membrane types at both flow conditions, but the number of different proteins was mostly highest over time on PMP membranes at the high flow rate. When comparing the heparin-coated PMP and PET membranes, it could be detected that higher flow rates led to increased adsorption of various proteins on both membrane types. Among the 10 most abundant proteins under all conditions were the proteins APOB, ALB, ATIII, FGG, FGA, FGB, APOE, and FN1. This is in part consistent with previous reports of protein layers detected on diverse biomaterials in which fibrinogen, albumin, vitronectin, and ApoA1 were found (31). The order of abundance is certainly modulated by the heparinization of the HFM. Many of these 10 most abundant proteins have a heparin-binding site (such as APOB, APOE, FN1, FCN2, and SERPINC1 ref. UniProt). Therefore, their presence on heparinized surfaces is to be expected. Considering their general relative abundance in blood, the order of the 10 most abundant proteins detected here could be expected as follows: ALB > TF > C3 > APOB > FN1 > Fibrinogen > IGHG1 > SERPINC1 > ITIH4 > APOE > FCN2 > LBp > ANG (32). Interestingly, in our study, the two proteins with the highest concentration in blood (albumin with 40 g/L and transferrin with 20 g/L), neither of which has a heparin-binding site, were adsorbed quite differently to the HFM membranes. Albumin was found on all membranes, whereas transferrin was only sporadically found among the first 10 proteins. Other proteins harbouring a heparin-binding site and are present in relatively high amounts in blood are e.g., SERPING1 and SERPIND1 (32), but these proteins were found on the membranes around the first 50–100 detected proteins. Of the two proteins with similar abundance in blood, APOB and C3 (32), APOB was found in higher amounts (often the most abundant protein) than C3, probably because APOB has a heparin-binding site. The binding of C3 to membranes, despite lacking a heparin binding site, is most likely due to its thioester bond exposed after activation (33, 34).

Although it has been reported that HMWK and HDL can displace fibrinogen (31), we could not detect such an effect in our study over 6 h. KNG1 was found among the first 42–62 detected proteins (once as the 19th most abundant protein on PMP membranes at 0.2 L/min) and proteins associated with HDL (such as the apolipoproteins A, C, D, E, F, M) were not found increased on the membranes over time. Only APOE was among the 10 most abundant proteins. Although we did not observe the described displacement, the order of abundance varied over time as described by Vroman (35, 36), and FGA consistently bound most abundantly on the PMP and PET membranes at a flow rate of 1 L/min.

Gore et al. (37) describe that the amount and reduction of plasma protein binding alone are not sufficient to improve hemocompatibility and hypothesize that specific antithrombin binding is mainly responsible for the improved hemocompatibility of the heparin coating. In our study, we also detected high ATIII (SERPINC1) binding under all measured conditions (rank 3–12). Importantly, a general observation was that lower flow rates resulted in less significant protein changes on the membranes than higher flow rates. More interestingly, PMP membranes showed less significant changes in proteins over 6 h than PET membranes.

The findings by Blezer et al. (38) underscore the dual nature of heparinized surfaces in their interaction with the coagulation system. While these surfaces are inherently thrombogenic, the immobilization of heparin combined with the plasma serine protease inhibitor antithrombin shifts their behavior towards strong antithrombotic activity by effectively slowing the propagation of blood clotting. This mechanism depends on the ability of the immobilized heparin-antithrombin complex to inhibit serine proteases such as thrombin. Thus, the effectiveness of heparinized surfaces in inhibiting the activation of serine proteases may also be influenced by diffusion processes. Specifically, the bioactivity of the heparinized surface could depend on how effectively its target molecules, such as serine protease inhibitors, diffuse to interact with heparin.

### 4.1 Influence of flow rate on protein binding

The process of thrombus formation and complement activation is initiated when blood comes into contact with artificial surfaces or damaged endothelium (39). The first step in this process is the adsorption of blood proteins, followed by the adhesion and activation of platelets. These processes are influenced by the fluid mechanics of the blood flow, in particular by the wall shear stress, which is the force per area of the wall caused by the flow. The constant wall shear stress in normal blood circulation indicates its important function (5). When blood comes into contact with a biomaterial, fluid mechanics, especially shear stress, can damage erythrocytes and platelets (40). Although experiments performed with blood plasma cannot accurately consider the effects of cell interaction with the surfaces, differential binding of plasma proteins was observed at different flow rates.

It was striking that an increased binding of the proteins of the coagulation cascade (PMP: VWF, F10, SERPIND1, F5; PET: VWF, F2) occurred especially at the late time point (360 min) at a high flow rate, while complement inhibitor proteins (VTN, CLU) were bound significantly more at the beginning (10 min, 1 L/min). Despite the commonly observed tendency of increased protein binding with increased flow rate, fibrinogen, F13B, and α-1-antitrypsin (SERPINA1) were found significantly more on PMP membranes at low flow rate. Increased fibrinogen deposition at a lower flow rate was observed previously (41). The simultaneous presence of both SERPINA1 and F13B is not surprising as α-1-antitrypsin is one of the most abundant proteins in fibrin clots (42) and the F13b subunit accelerates the crosslinking of fibrin by binding to FXIII-A, fibrinogen, and thrombin (43). Shear stress caused by blood flow can trigger the activation of von Willebrand factor (vWF) and its binding to the platelet glycoprotein Ib receptor (GPIb), followed by activation of GPIIb/IIIa receptor, which binds vWF or fibrinogen and mediates platelet aggregation (44). Thus, high shear stress and the resulting conformational changes in vWF could increase its binding to the HFM surfaces, as observed *in vitro* with isolated platelets subjected to increased shear stress. When platelets were suspended in HEPES-buffer alone, elevated shear stress created in stainless steel viscosimeter with rotating cone and stationary plate led to increased platelet binding. However, the addition of fibrinogen, vWF multimeric forms, or both, significantly increased shear-induced platelet adhesion (45). On the PET membrane, in addition to the increased binding of coagulation proteins at a higher flow rate, there was also increased binding of complement proteins (C4A; C9; CFB; C8B; Supplementary Figure S1) at some time points. The significantly increased sC5b-9 levels after the incubation of blood with the PET membrane for 360 min at a high flow rate compared to the low flow rate also indicate a possible correlation with the increased binding of complement proteins to the membranes. Our studies with whole blood also showed that high flow rates led to increased activation of platelets, neutrophils, and the complement system, resulting in a deterioration of hemocompatibility. Medium flow rates for long-time ECMO are therefore desirable for reducing the protein adsorption to the HFM as well as reducing shear stress to cell components (46). This however, is only possible for some patients as the arterial oxygen saturation of 80% or higher is the main goal of ECMO (47) and the flow rates have to be adjusted accordingly.

### 4.2 Influence of membrane type on protein binding

The influence of the membrane types was only significant for very few proteins, especially at the low flow rate. This indicates that the flow rate has a greater influence on the adsorption of plasma proteins. Nevertheless, the influence of the material should not be underestimated. PMP and PET are both thermoplastic materials commonly used in medical devices and other applications (48, 49). However, they have some differences in their properties and characteristics. PMP is an amorphous polymer composed of repeating units of methylpentene monomers (50), while PET is a semi-crystalline polymer composed of repeating units of ethylene glycol and terephthalic acid (51). The comparison of the adsorbed plasma proteins on both materials showed that the fibrinogen (FGG, FGB, and FGA) and some other proteins associated with the inhibition of coagulation (SERPIND1) and fibrinolysis (SERPINF2) bind significantly more frequently to the PET membrane than to the PMP membrane at a high flow rate. Proteins of the complement system (MASP1, FCN3), but also of the coagulation cascade (VWF) were more frequently bound to the PMP membrane at high flow. This indicates that at high flow rates, PET HFM seems to have more influence on coagulation activation, whereas complement activation seems to be more prominent for PMP HFM. Under low flow conditions, two inhibitory proteins, C1-esterase inhibitor (SERPING1) and alpha-1-antitrypsin (SERPINA1), showed increased binding to the PMP membrane at 90 min, indicating that their presence could help to mitigate complement activation and inflammation. In a previous study, heparin-coating of PMP HFM not only reduced the overall number of adsorbed proteins compared to non-coated PMP HFM but also significantly decreased adsorption of coagulation-related proteins (FXII, FIX, FV, FX, fibrinogen, kalikrein, kininogen) and complement activation- related proteins (C3, C4, FH) (24). On the other hand, some proteins with heparin-binding sites [FXI, ATIII (=SERPINC1), C1INH (=SERPING1)] were more abundant on heparin-coated PMP HFM. We could therefore conclude that the heparin-coating generally led to reduced protein adsorption, particularly for proteins involved in coagulation and complement activation, while enhancing the binding of proteins that inhibit these processes. Compared to the present study, where both membrane types were heparin-coated, some similarities were observed: (1) for both heparin-coated membrane types, the number of adsorbed proteins present at all time points were reasonably similar; (2) activation-related proteins previously shown to bind more to non-coated PMP HFM did not show significant differences in binding between heparin-coated PMP and PET membranes; (3) levels of C1INH (SERPING1), ATIII (SERPINC1), and FXI were also not significantly different between heparin-coated PMP and PET HFM. Thus, heparinization of both membrane types has likely reduced overall protein adsorption and decreased binding of coagulation and complement activation-related proteins. When assessing hemocompatibility and developing new coating approaches, the literature often only considers the gas exchange membrane and neglects the heat exchange membrane (52–54). Our findings indicate that this approach is acceptable if the heat exchange membrane is also heparinized, as plasma protein adsorption on both membrane types was relatively similar and the flow rate played the most decisive role. Especially, considering increased presence of C1INH (SERPING1) and alpha-1-antitrypsin (SERPINA1) on PMP HFM at the lower flow rate.

## 5 Conclusion

In this study, the binding of plasma proteins to the surfaces of heparin-coated PMP or PET membranes was investigated at low and high flow rates over 6 h using miniature devices, and the adsorbed proteins on the membranes were measured by MS. The adsorption of plasma proteins on both membrane types was relatively similar, which could be related to the heparin-coating of both membrane materials. In contrast, the flow rate played the most decisive role. The main differences in protein binding occurred at early time points and higher flow rate and were no longer present after 6 h at a low flow rate. The studies with whole blood also showed an increased activation of thrombocytes, inflammation, and complement system at high flow rate for both material types compared to low flow rate. The main differences between materials observed were the increased binding of coagulation-associated proteins on PET HFM and complement activation-associated proteins on PMP HFM at the higher flow rate. Notably, at a low flow rate, PMP HFM exhibited a significant increase in binding of complement and inflammation inhibitors, suggesting a potential benefit of lowering the flow rate apart from the general reduction in protein adsorption.

## Data Availability

The original contributions presented in the study are included in the article/Supplementary Material, further inquiries can be directed to the corresponding author.

## Supplementary material

Supplementary Figure 1 Binding profiles of complement components with differences between the materials and flow rates. Statistical analysis was performed using unpaired two-tailed *t*-test to determine the differences between heparin-coated PMP membrane and heparin-coated PET membrane, and the differences between 1 L/min and 0.2 L/min flow rate. Shown are mean values ± SD, *n* = 3; * *p* < 0.05; ** *p* < 0.01.

Supplementary Figure 2 Binding profiles of coagulation components with differences between the materials and flow rates. Statistical analysis was performed using unpaired two-tailed *t*-test to determine the differences between heparin-coated PMP membrane and heparin-coated PET membrane, and the differences between 1 L/min and 0.2 L/min flow rate. Shown are mean values ± SD, *n* = 3; * *p* < 0.05; ** *p* < 0.01.
