# Supplementary figures and images for: Comparative study of flow rate- and material-dependent human plasma protein adsorption on oxygenator membranes and heat exchanger materials
Source: Front Cardiovasc Med. 2025 Jun 17;12:1578538. doi: 10.3389/fcvm.2025.1578538 (PMC12211862; doi:10.3389/fcvm.2025.1578538)

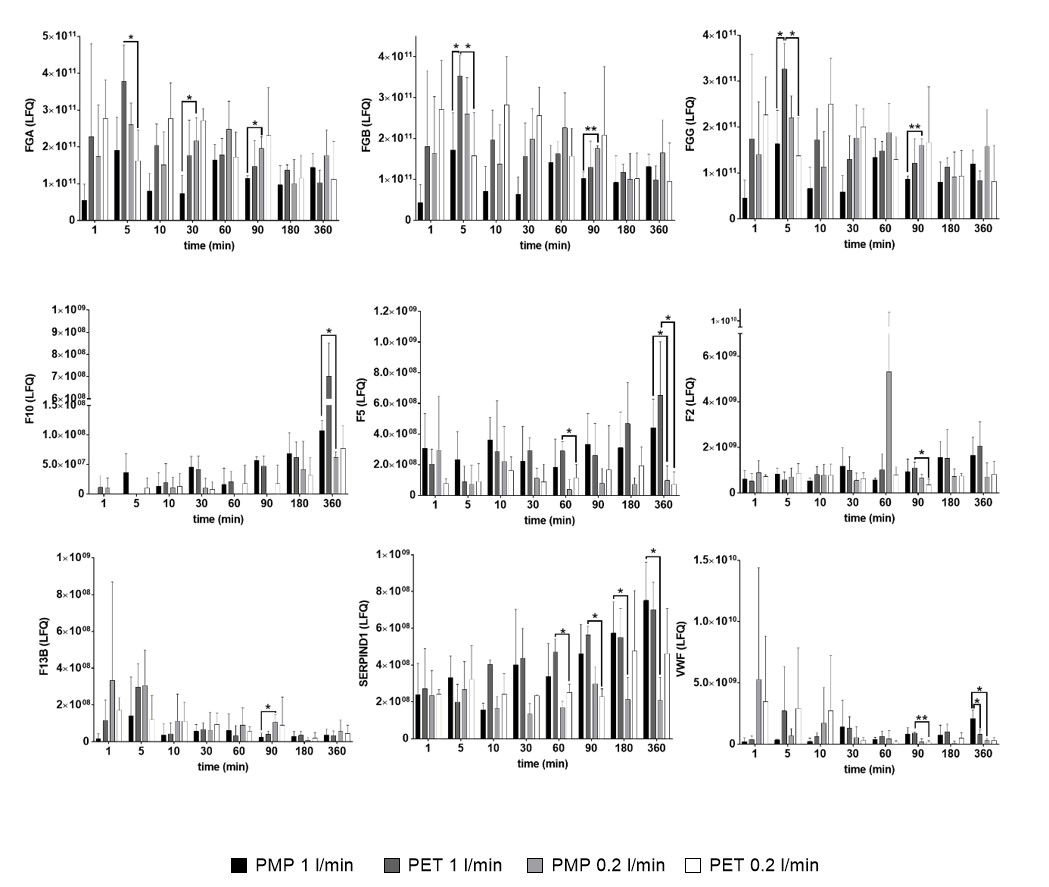

Supplement: Supplementary Figure 1 — Binding profiles of complement components with differences between the materials and flow rates. Statistical analysis was performed using unpaired two-tailed t-test to determine the differences between heparin-coated PMP membrane and heparin-coated PET membrane, and the differences between 1 L/min and 0.2 L/min flow rate. Shown are mean values ± SD, n = 3; * p < 0.05; ** p < 0.01. [file Image1.jpeg]

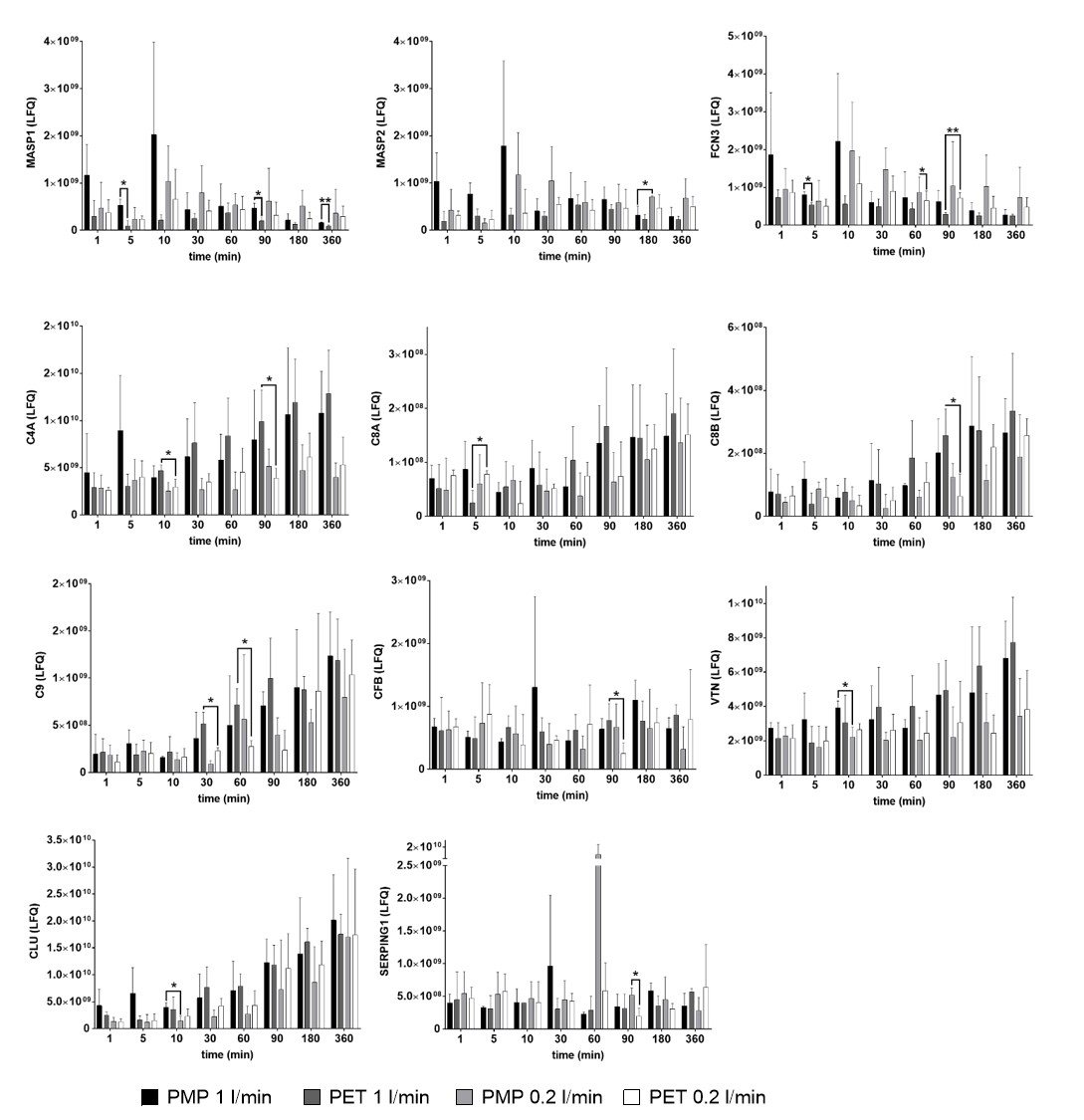

Supplement: Supplementary Figure 2 — Binding profiles of coagulation components with differences between the materials and flow rates. Statistical analysis was performed using unpaired two-tailed t-test to determine the differences between heparin-coated PMP membrane and heparin-coated PET membrane, and the differences between 1 L/min and 0.2 L/min flow rate. Shown are mean values ± SD, n = 3; * p < 0.05; ** p < 0.01. [file Image2.jpeg]
