# Supplementary material for: Comparative study of flow rate- and material-dependent human plasma protein adsorption on oxygenator membranes and heat exchanger materials
Source: Front Cardiovasc Med. 2025 Jun 17;12:1578538. doi: 10.3389/fcvm.2025.1578538 (PMC12211862; doi:10.3389/fcvm.2025.1578538)
Supplement: Supplementary file 3 [file Table1.pdf]

Supplementary Table 1: Complete list of all measured proteins, sorted by frequency on the heparin-coated PMP membrane with a flow rate of 1 l/min at the indivdual time points after plasma contact (1-360min). Colored fields show significant changes (over time, between materials, or flow rates) according to the legend below.

		# of most abundance desorbed from minature devices - PMP 1 l/min							
**Protein names**	**Gene names**	**1 min**	**5 min**	**10 min**	**30 min**	**60 min**	**90 min**	**180 min**	**360 min**
Apolipoprotein B-100;Apolipopr	APOB	1	5	1	2	3	1	2	4
Serum albumin	ALB	2	1	2	1	5	3	1	5
Antithrombin-III	SERPINC1	3	8	5	6	6	6	9	9
Fibrinogen alpha chain;Fibrinop	FGA	4	2	3	3	1	2	3	1
Fibrinogen gamma chain	FGG	5	4	6	5	4	5	5	3
Fibrinogen beta chain;Fibrinope	FGB	6	3	4	4	2	4	4	2
Apolipoprotein E	APOE	7	7	7	7	8	7	10	10
Lipopolysaccharide-binding pro	LBP	8	13	10	17	12	13	16	12
Ficolin-2	FCN2	9	10	9	14	11	12	15	16
Fibronectin;Anastellin;Ugl-Y1;U	FN1	10	6	8	10	7	9	8	7
Complement C3;Complement C	C3	11	9	12	8	9	8	7	8
Complement C1q subcompone	C1QC	12	22	14	23	17	20	23	20
Complement C1q subcompone	C1QB	13	24	16	24	16	19	22	21
Apolipoprotein(a)	LPA	14	28	11	18	14	11	19	15
Apolipoprotein A-I;Proapolipopr	APOA1	15	15	15	11	18	15	17	18
Angiogenin	ANG	16	17	13	15	13	17	28	25
Coagulation factor XI;Coagulati	F11	17	39	18	28	23	26	34	32
Serotransferrin	TF	18	19	23	9	19	22	12	24
Ig gamma-1 chain C region	IGHG1	19	12	19	12	15	16	11	14
Complement C1q subcompone	C1QA	20	34	24	36	26	30	31	30
Complement C1r subcomponen	C1R	21	27	20	29	28	33	44	45
Ig mu chain C region	IGHM	22	16	17	21	21	21	26	27
Complement C1s subcompone	C1S	23	31	22	35	32	34	47	48
Inter-alpha-trypsin inhibitor hea	ITIH4	24	11	21	20	10	10	6	6
Alpha-2-macroglobulin	A2M	25	21	28	13	24	25	14	23
Alpha-1-antitrypsin;Short peptic	SERPINA1	26	23	33	16	25	28	20	26
Complement C4-A;Complemen	C4A	27	14	29	22	22	18	18	13
Clusterin;Clusterin beta chain;C	CLU	28	18	30	25	20	14	13	11
Lysozyme C;Lysozyme	LYZ	29	45	25	38	33	40	64	67
Ig kappa chain C region	IGKC	30	20	32	26	27	29	21	29
Vitronectin;Vitronectin V65 subu	VTN	31	25	31	31	29	27	25	19
Ig alpha-1 chain C region	IGHA1	32	29	37	27	34	31	27	34
Proteoglycan 4;Proteoglycan 4	PRG4	33	36	35	32	36	81	97	72
Complement factor H	CFH	34	41	42	42	43	38	39	50
Haptoglobin;Haptoglobin alpha	HP	35	38	45	19	35	36	24	41
Ig gamma-3 chain C region	IGHG3	36	33	34	41	37	32	29	35
Ficolin-3	FCN3	37	49	36	68	48	58	71	84
Plasma serine protease inhibito	SERPINA5	38	40	27	50	30	24	40	22
Apolipoprotein A-IV	APOA4	39	30	44	33	44	37	37	31
Ribonuclease 4	RNASE4	40	57	40	55	41	49	88	78
Immunoglobulin lambda-like po	IGLL5;IGLC1	41	32	43	34	39	35	32	38
Ig gamma-2 chain C region	IGHG2	42	37	39	39	42	42	33	46
Hyaluronan-binding protein 2;H	HABP2	43	35	26	46	31	23	43	17
Inter-alpha-trypsin inhibitor hea	ITIH2	44	42	53	43	38	39	30	28
Leukocyte cell-derived chemota	LECT2	45	74	46	64	51	78	123	112
Hemopexin	HPX	46	51	58	30	45	46	36	59
Retinoic acid receptor responde	RARRES2	47	100	47	72	46	59	100	88
Mannan-binding lectin serine pr	MASP1 heavy	48	58	38	78	58	69	99	102
Apolipoprotein C-I;Truncated ap	APOC1	49	53	49	59	55	54	81	74
Kininogen-1;Kininogen-1 heavy	KNG1	50	52	56	48	47	48	45	47
Cadherin-1;E-Cad/CTF1;E-Cad	CDH1	51	68	50	75	56	75	104	106
Mannan-binding lectin serine pr	MASP2	52	54	41	82	49	52	83	83
Insulin-like growth factor-bindin	IGFBP3	53	91	52	89	57	56	96	89
Plasminogen;Plasmin heavy ch	PLG	54	26	54	45	40	41	35	33
Apolipoprotein C-III	APOC3	55	43	48	58	50	61	80	70
C4b-binding protein alpha chair	C4BPA	56	56	62	57	66	53	56	54
Apolipoprotein C-II;Proapolipop	APOC4-APOC	57	44	55	91	60	51	89	86
Chondroadherin	CHAD	58	83	51	70	53	74	113	113
Apolipoprotein D	APOD	59	89	61	74	69	67	73	75
Insulin-like growth factor-bindin	IGFBP5	60	92	57	81	72	90	138	140
Ceruloplasmin	CP	61	66	72	37	68	72	38	73
Complement factor B;Complem	CFB	62	59	67	49	64	55	48	55
Vitamin D-binding protein	GC	63	67	76	40	61	66	46	81
Inter-alpha-trypsin inhibitor hea	ITIH1	64	69	69	53	62	63	50	53
Prothrombin;Activation peptide	F2	65	48	64	52	54	45	41	39
Apolipoprotein A-II;Proapolipop	APOA2	66	55	63	60	78	65	63	66
Lactotransferrin;Lactoferricin-H	LTF	67	113	60	77	81	106	151	147
Hemoglobin subunit beta;LVV-I	HBB	68	46	66	76	96	76	74	82

Serum amyloid P-component;S	APCS	69	62	68	73	77	57	60	40
Alpha-1-antichymotrypsin;Alpha	SERPINA3	70	61	83	51	65	68	49	60
Beta-2-glycoprotein 1	APOH	71	84	85	63	83	87	67	101
Stromal cell-derived factor 1;SD	CXCL12	72	77	59	144	107	98	107	104
Plasma protease C1 inhibitor	SERPING1	73	78	70	56	84	83	61	71
Serum paraoxonase/arylesteras	PON1	74	94	71	80	75	79	72	69
Ribonuclease pancreatic	RNASE1	75	82	65	79	63	95	122	111
Alpha-2-antiplasmin	SERPINF2	76	47	78	62	52	44	42	37
Protein AMBP;Alpha-1-microglo	AMBP	77	85	81	85	86	91	77	80
Transthyretin	TTR	78	87	92	66	88	94	76	98
Alpha-2-HS-glycoprotein;Alpha-	AHSG	79	70	87	54	73	77	58	56
Angiotensinogen;Angiotensin-1	AGT	80	63	90	65	71	73	55	63
Thrombospondin-4	THBS4	81	139	82	110	99	147	193	169
Coagulation factor V;Coagulatio	F5	82	90	75	98	93	86	84	64
Histidine-rich glycoprotein	HRG	83	75	100	71	89	109	78	99
	IGHV3-72	84	65	74	86	97	92	79	96
Alpha-1-acid glycoprotein 1	ORM1	85	97	124	44	80	104	59	132
Serum amyloid A-4 protein	SAA2-SAA4;S	86	95	96	117	95	89	102	87
Tetranectin	CLEC3B	87	126	84	104	91	108	129	120
Ig gamma-4 chain C region	IGHG4	88	73	95	67	85	82	53	62
Complement factor H-related pr	CFHR1	89	98	77	105	98	84	103	91
Complement C5;Complement C	C5	90	64	104	69	76	60	54	49
Matrix Gla protein	MGP	91	138	91	189	90	99	137	115
CD5 antigen-like	CD5L	92	88	79	95	100	93	101	103
Heparin cofactor 2	SERPIND1	93	76	108	83	74	70	62	52
Antileukoproteinase	SLPI	94	0	89	103	114	158	0	0
Apolipoprotein L1	APOL1	95	99	101	97	87	85	66	51
Procollagen C-endopeptidase e	PCOLCE	96	188	86	133	110	117	153	134
Hemoglobin subunit alpha	HBA1;HBA2	97	81	107	100	147	116	117	128
Complement component C9;Co	C9	98	79	105	87	59	50	52	43
von Willebrand factor;von Willel	VWF	99	72	93	47	70	47	57	36
Alpha-1B-glycoprotein	A1BG	100	101	125	61	101	112	69	109
Complement factor H-related pr	CFHR5	101	96	116	152	151	71	70	68
C-C motif chemokine 18;CCL18	CCL18	102	176	103	176	125	146	198	213
Properdin	CFP	103	86	159	168	165	64	119	168
C-C motif chemokine 14;HCC-1	CCL14	104	172	123	179	128	177	0	0
Procollagen C-endopeptidase e	PCOLCE2	105	167	88	131	108	132	192	172
	IGHV3-15	106	132	98	118	103	110	124	141
Ig kappa chain V-II region FR	IGKV2D-28	107	102	129	88	116	137	93	124
Immunoglobulin J chain	IGJ;JCHAIN	108	105	113	119	112	107	134	139
Alpha-1-antitrypsin;Short peptic	SERPINA1	109	157	134	124	163	153	111	151
Apolipoprotein M	APOM	110	148	132	132	129	125	143	135
Ig heavy chain V-III region BUT	IGHV3-74;IGH	111	117	73	84	79	88	68	93
Semenogelin-1;Alpha-inhibin-92	SEMG1	112	50	94	94	115	0	0	0
Myosin-9	MYH9	113	151	97	157	154	144	184	201
Apolipoprotein C-IV	APOC4	114	155	122	0	150	138	187	165
Tenascin-X	TNXB	115	0	155	259	0	183	0	0
Glyceraldehyde-3-phosphate de	GAPDH	116	104	110	113	113	114	131	123
Apolipoprotein A-V	APOA5	117	168	121	155	140	121	139	107
Cholesteryl ester transfer protei	CETP	118	80	114	112	67	43	51	42
Phospholipid transfer protein	PLTP	119	119	117	150	104	100	91	65
Afamin	AFM	120	170	161	96	132	157	108	152
Monocyte differentiation antigen	CD14	121	165	102	147	134	145	148	136
Pregnancy zone protein	PZP	122	147	143	137	105	124	86	130
Carboxypeptidase N catalytic c	CPN1	123	129	99	116	121	123	140	133
Complement C4-B;Complemen	C4B	124	93	80	99	94	102	92	92
Ectonucleotide pyrophosphatas	ENPP2	125	0	119	200	152	164	208	219
Extracellular matrix protein 1	ECM1	126	115	115	139	82	169	165	148
Complement component C8 be	C8B	127	114	141	127	109	101	87	85
Zinc-alpha-2-glycoprotein	AZGP1	128	123	145	93	130	167	146	182
Kallistatin	SERPINA4	129	136	149	123	142	122	105	97
Cathelicidin antimicrobial peptic	CAMP	130	144	137	173	191	155	201	202
Fibulin-1	FBLN1	131	103	138	151	106	96	82	61
Retinol-binding protein 4;Plasm	RBP4	132	142	164	107	176	154	136	161
Haptoglobin-related protein	HPR	133	124	130	115	122	127	127	116
Ig kappa chain V-I region AU;Ig	kappa chain V	134	116	127	101	118	119	112	114
	IGHV3-49	135	130	139	181	0	159	144	142
Complement component C8 alp	C8A	136	121	148	134	133	115	115	105
Actin, cytoplasmic 1;Actin, cyto	ACTB	137	110	136	102	102	80	85	58
Complement factor D	CFD	138	0	111	161	111	149	152	154

Pleckstrin	PLEK	139	162	109	143	119	141	167	191
Coagulation factor XIII A chain	F13A1	140	71	106	128	92	103	110	95
Prenylcysteine oxidase 1	PCYOX1	141	0	120	180	131	130	179	155
Coagulation factor XII;Coagulat	F12	142	175	169	114	172	161	141	143
Ig kappa chain V-IV region	IGKV4-1	143	108	128	106	155	120	106	126
Ig kappa chain V-III region B6	IGKV3-20	144	0	112	92	143	118	95	138
Plasma kallikrein;Plasma kallikr	KLKB1	145	178	163	108	170	165	132	150
Complement component C7	C7	146	109	167	121	127	97	75	76
Nephronectin	NPNT	147	0	131	153	139	176	207	210
Complement component C6	C6	148	112	152	122	120	111	98	90
Corticosteroid-binding globulin	SERPINA6	149	166	174	120	138	150	120	131
N-acetylmuramoyl-L-alanine am	PGLYRP2	150	135	147	111	135	126	121	108
Ig heavy chain V-II region NEW	IGHV4-61	151	154	0	0	167	163	149	157
Alpha-1-acid glycoprotein 2	ORM2	152	158	0	90	145	178	142	197
Vitamin K-dependent protein S	PROS1	153	145	157	140	173	129	109	94
Insulin-like growth factor-bindin	IGFALS	154	152	150	126	141	143	126	118
Collagen alpha-1(XVIII) chain;E	COL18A1	155	0	146	255	198	216	0	0
	IGHV5-51	156	150	172	138	164	171	150	171
Fatty acid-binding protein, epide	FABP5	157	146	166	196	174	205	0	0
Phospholipase A2, membrane a	PLA2G2A	158	0	158	178	0	185	206	193
Complement component C8 ga	C8G	159	128	165	142	162	128	118	110
Caspase-8;Caspase-8 subunit p	CASP8	160	0	0	0	0	0	0	0
Hepatocyte growth factor activa	HGFAC	161	0	175	232	0	0	233	0
Cystatin-A;Cystatin-A, N-termin	CSTA	162	106	144	162	146	0	0	0
	IGHV3OR16-9	163	133	170	216	0	142	154	166
Histone H4	HIST1H4A	164	122	140	184	199	152	164	153
Neutrophil defensin 3;HP 3-56;I	DEFA3;DEFA1	165	0	182	164	0	0	158	0
Ig lambda chain V-III region SH	IGKV3-19	166	0	0	198	157	134	135	119
Histone H2A type 1-J;Histone H	HIST1H2AJ;H	167	111	118	154	156	182	209	0
Signal peptide, CUB and EGF-l	SCUBE2	168	0	133	145	0	203	221	0
Ig lambda chain V-I region HA	IGLV1-44	169	156	183	0	168	186	0	0
Ig lambda chain V-IV region Hil	IGLV3-25	170	125	0	146	148	156	147	185
EGF-containing fibulin-like extra	EFEMP1	171	120	153	183	117	113	94	79
Selenoprotein P	SEPP1	172	0	188	190	0	192	189	206
Platelet factor 4;Platelet factor 4	PF4;PF4V1	173	0	135	174	160	199	0	209
Pigment epithelium-derived fact	SERPINF1	174	185	219	129	189	207	157	177
Carboxypeptidase N subunit 2	CPN2	175	173	173	141	166	148	130	127
Arginase-1	ARG1	176	137	228	219	177	0	0	0
Inter-alpha-trypsin inhibitor hea	ITIH3	177	174	234	165	137	139	114	100
Serum amyloid A-2 protein	SAA2	178	0	0	0	159	162	174	0
Dermcidin;Survival-promoting p	DCD	179	160	176	193	180	198	0	0
Glutathione peroxidase;Glutath	GPX3	180	153	156	169	123	105	90	57
Complement C1q tumor necros	C1QTNF3-AM	181	0	225	236	0	0	0	0
Transforming growth factor-beta	TGFBI	182	163	201	248	169	160	133	129
Polyubiquitin-C;Ubiquitin;Ubiqu	UBC;UBB;RPS	183	0	0	175	0	0	0	0
Annexin A2;Annexin;Putative ar	ANXA2;ANXA2	184	131	187	192	171	0	0	0
Desmoglein-1	DSG1	185	0	226	221	190	0	0	0
Ig delta chain C region	IGHD	186	180	229	0	209	188	197	188
Collagen alpha-1(I) chain	COL1A1	187	0	184	225	0	0	0	0
Mannan-binding lectin serine pr	MASP1 light c	188	0	0	0	0	0	0	0
Olfactomedin-like protein 1	OLFML1	189	0	210	0	0	0	0	0
Protein S100-A8;Protein S100-A	S100A8	190	140	189	136	124	197	0	0
Coagulation factor XIII B chain	F13B	191	107	160	156	126	175	169	164
Collectin-11	COLEC11	192	0	224	0	0	0	0	0
Cartilage intermediate layer pro	CILP	193	0	200	0	0	0	0	0
Alpha-enolase;Enolase	ENO1	194	0	194	0	0	0	0	0
Prostaglandin-H2 D-isomerase	PTGDS	195	0	0	0	0	0	0	0
Zymogen granule protein 16 ho	ZG16B	196	0	205	205	0	168	0	0
Olfactomedin-like protein 3	OLFML3	197	0	193	223	0	0	0	0
Histone H2B type 1-L;Histone H	HIST1H2BL;H	198	127	154	171	0	204	0	199
Elongation factor 1-alpha 1;Puta	EEF1A1;EEF1	199	0	186	240	187	209	227	0
DnaJ homolog subfamily C mem	DNAJC3	200	0	191	0	195	211	0	0
	IGHV1-69-2	201	0	0	0	188	195	0	0
Tissue factor pathway inhibitor	TFPI	202	0	177	203	207	234	0	0
	IGLV8-61	203	0	0	0	0	0	171	200
Semenogelin-2	SEMG2	204	60	181	167	136	0	0	0
Platelet basic protein;Connectiv	PPBP	205	0	0	0	0	0	0	0
Alcohol dehydrogenase 4	ADH4	206	0	202	0	0	191	0	0
Laminin subunit alpha-4	LAMA4	207	0	221	0	0	0	0	0
Insulin-like growth factor II;Insu	IGF2	208	0	218	0	0	0	0	0

Caldesmon	CALD1	209	0	180	0	0	0	0	0
Beta-galactoside alpha-2,6-sial	ST6GAL1	210	0	0	0	0	0	0	0
Myeloperoxidase;Myeloperoxid	MPO	211	0	179	204	0	237	0	0
Protein-glutamine gamma-gluta	TGM3	212	118	212	194	200	0	0	0
Carboxypeptidase B2	CPB2	213	169	216	201	197	222	199	176
Junction plakoglobin	JUP	214	0	0	0	0	0	0	0
Thyroxine-binding globulin	SERPINA7	215	0	0	172	196	219	161	0
Fructose-1,6-bisphosphatase 1	FBP1	216	0	0	263	205	189	0	0
Collagen alpha-2(I) chain	COL1A2	217	0	192	0	0	0	0	0
Lumican	LUM	218	0	0	188	0	238	182	195
Latent-transforming growth fact	LTBP2	219	0	0	0	0	0	0	0
Deoxyribonuclease gamma;De	DNASE1L3	220	0	162	218	186	206	228	0
Talin-1	TLN1	221	0	0	256	203	235	225	186
Phosphatidylinositol-glycan-spe	GPLD1	222	197	0	210	0	194	183	187
Spondin-1	SPON1	223	0	0	0	0	0	0	0
Serpin B3	SERPINB3	224	159	230	158	158	0	0	0
Protein S100-A7;Protein S100-	S100A7;S100	225	143	203	187	182	236	0	0
Polymeric immunoglobulin rece	PIGR	226	171	231	109	208	246	0	0
Bactericidal permeability-increa	BPI	227	0	151	211	201	230	0	0
Inter-alpha-trypsin inhibitor hea	ITIH5	228	0	0	0	0	0	0	0
Complement factor I;Compleme	CFI	229	0	0	130	0	0	190	0
Desmoplakin	DSP	230	0	0	0	0	0	0	0
Bone morphogenetic protein 1	BMP1	231	0	190	224	214	215	224	214
Histone H3;Histone H3.3C;Hist	HIST2H3PS2;	232	0	0	229	0	0	0	0
Angiopoietin-related protein 4	ANGPTL4	233	0	213	0	0	0	0	0
C-reactive protein;C-reactive pr	CRP	234	0	222	182	149	133	175	117
Pyruvate kinase PKM;Pyruvate	PKM	235	0	0	0	0	247	215	181
Apolipoprotein F	APOF	236	0	0	270	0	0	231	0
Collagen alpha-2(XI) chain	COL11A2	237	0	196	253	0	0	0	0
Multimerin-1;Platelet glycoprote	MMRN1	238	187	185	215	206	0	0	0
Secreted frizzled-related protei	FRZB	239	0	0	0	0	0	0	0
Complement C2;Complement C	C2	240	0	0	246	0	0	0	0
Prolactin-inducible protein	PIP	241	181	207	197	202	190	0	0
Chymotrypsin-C	CTRC	242	0	0	0	0	0	0	0
Phospholipase A1 member A	PLA1A	243	0	0	0	0	0	0	0
BPI fold-containing family A me	BPIFA1	244	194	0	213	212	0	0	0
Sushi repeat-containing protein	SRPX	245	0	238	260	0	0	0	0
Inter-alpha-trypsin inhibitor hea	ITIH4	0	0	0	0	0	62	65	44
Tsukushin	TSKU	0	0	208	206	161	136	125	77
Coagulation factor X;Factor X li	F10	0	161	195	166	179	151	145	121
Integrin alpha-IIb;Integrin alpha	ITGA2B	0	0	0	235	0	170	168	122
Band 3 anion transport protein	SLC4A1	0	0	0	160	175	135	156	125
Flavin reductase (NADPH)	BLVRB	0	0	0	0	0	0	176	137
Ig kappa chain V-III region VG	IGKV3D-11	0	0	0	125	0	140	116	144
Fermitin family homolog 3	FERMT3	0	0	0	252	213	174	210	145
Fibronectin;Anastellin;Ugl-Y1;U	FN1	0	134	126	0	0	131	0	146
Sex hormone-binding globulin	SHBG	0	0	0	212	0	208	160	149
Protein Z-dependent protease i	SERPINA10	0	0	0	217	0	172	172	156
Coagulation factor VII;Factor VI	F7	0	0	0	0	0	184	194	158
Ig heavy chain V-III region CAM	IGHV3-23	0	149	168	163	144	166	163	159
Collagen alpha-3(VI) chain	COL6A3	0	0	0	265	194	180	173	160
Coagulation factor IX;Coagulati	F9	0	191	0	195	0	218	177	162
Peroxiredoxin-6	PRDX6	0	0	0	0	211	202	178	163
Sulfhydryl oxidase 1	QSOX1	0	192	235	220	210	187	196	167
Peptidyl-glycine alpha-amidatin	PAM	0	0	0	0	192	212	181	170
Integrin beta;Integrin beta-3	ITGB3	0	0	0	0	0	213	204	173
Lipoprotein lipase	LPL	0	193	178	202	0	181	188	174
Phosphatidylcholine-sterol acyl	LCAT	0	0	0	257	0	217	195	175
Alpha-1,3-mannosyl-glycoprote	MGAT1	0	0	0	0	204	214	212	178
Actin, alpha skeletal muscle;Ac	ACTA1;ACTC	0	0	0	0	0	0	185	179
Fetuin-B	FETUB	0	0	0	0	0	220	180	180
78 kDa glucose-regulated prote	HSPA5	0	189	0	0	0	0	220	183
Secreted phosphoprotein 24	SPP2	0	183	220	0	0	196	205	184
Ras-related protein Rap-1b;Ra	RAP1B;RAP1	0	0	0	0	0	0	217	189
C4b-binding protein beta chain	C4BPB	0	0	0	247	0	229	211	190
Inter-alpha-trypsin inhibitor hea	ITIH4	0	0	0	0	0	231	216	192
Fibrinogen-like protein 1	FGL1	0	0	0	0	181	0	0	194
Galectin-3-binding protein	LGALS3BP	0	0	0	199	0	239	202	196
14-3-3 protein zeta/delta	YWHAZ	0	0	0	0	0	0	0	198
Ig kappa chain V-I region Daud	IGKV1-6;IGKV	0	186	0	185	178	193	170	203

Erythrocyte band 7 integral mer	STOM	0	0	0	0	0	0	0	204
Vitamin K-dependent protein C;	PROC	0	0	0	0	0	228	213	205
Serum amyloid A-1 protein;Amy	SAA1	0	0	233	261	184	0	203	207
GTP-binding nuclear protein Ra	RAN	0	190	199	243	0	200	222	208
Hyaluronidase-1	HYAL1	0	0	0	0	0	233	219	211
Coagulation factor VIII;Factor V	F8	0	0	0	0	0	0	0	212
Spectrin beta chain, erythrocyti	SPTB	0	0	0	0	0	227	0	215
Tetraspanin;CD9 antigen	CD9	0	0	0	0	0	0	0	216
N-acetylglucosamine-1-phosph	GNPTG	0	0	0	0	0	0	223	217
Basement membrane-specific h	HSPG2	0	0	0	0	0	0	230	218
Fibulin-2	FBLN2	0	0	0	0	0	0	0	220
HLA class I histocompatibility a	HLA-B;HLA-C;	0	0	0	0	0	0	0	221
Transforming growth factor beta	TGFBR3	0	0	0	0	0	0	234	222
Tryptophan--tRNA ligase, cytop	WARS	0	0	0	0	0	0	0	223
Serine protease HTRA1	HTRA1	0	0	0	0	0	0	0	224
Putative heat shock 70 kDa pro	HSPA7;HSPA(	0	0	0	0	0	0	0	225
Dihydropyrimidinase-related pro	DPYSL3;CRM	0	0	0	0	0	0	128	0
Leucine-rich alpha-2-glycoprote	LRG1	0	0	0	135	0	0	155	0
Ig heavy chain variable region 1	IGHV1-18	0	0	0	159	0	0	159	0
	IGKV2-24;IGK	0	0	0	177	0	0	162	0
Ig lambda chain V-III region LOI		0	0	0	0	0	0	166	0
Ig kappa chain V-I region HK10	IGKV1-5	0	0	0	191	0	0	186	0
	IGKV1-27	0	0	237	0	0	225	191	0
Ig heavy chain V-I region HG3		0	0	0	0	0	0	200	0
	IGKV3D-15	0	0	0	228	0	0	214	0
Ig lambda chain V-VI region AR		0	0	0	0	0	0	218	0
Multimerin-2	MMRN2	0	0	0	0	0	0	226	0
Insulin-like growth factor I	IGF1	0	0	204	231	193	0	229	0
	IGHV1-24	0	0	0	0	0	0	232	0
Alpha-mannosidase 2	MAN2A1	0	0	0	0	0	0	235	0
Ig heavy chain V-I region V35		0	0	0	0	0	0	236	0
Alpha-amylase 2B;Pancreatic a	AMY2B;AMY2	0	177	0	186	183	173	0	0
Eosinophil cationic protein	RNASE3	0	0	142	237	185	179	0	0
Ankyrin-1	ANK1	0	0	0	251	0	201	0	0
Asporin	ASPN	0	0	206	0	0	210	0	0
Ig heavy chain V-III region DOB		0	0	0	234	0	221	0	0
Tartrate-resistant acid phospha	ACP5	0	0	198	0	0	223	0	0
BPI fold-containing family B me	BPIFB1	0	0	227	244	0	224	0	0
Cystatin-S;Cystatin-SA;Cystatin	CST4;CST2;C	0	0	0	242	0	226	0	0
Protein S100-A9	S100A9	0	141	171	149	153	232	0	0
Deleted in malignant brain tumo	DMBT1	0	0	0	0	0	240	0	0
Protein 4.1	EPB41	0	0	0	0	0	241	0	0
Heat shock protein beta-1	HSPB1	0	0	0	0	0	242	0	0
BPI fold-containing family A me	BPIFA2	0	0	0	0	0	243	0	0
Hepatic triacylglycerol lipase	LIPC	0	0	209	0	0	244	0	0
Adipocyte plasma membrane-a	APMAP	0	0	0	0	0	245	0	0
Caspase-14;Caspase-14 subur	CASP14	0	199	0	238	215	0	0	0
Lipocalin-1	LCN1	0	164	0	148	0	0	0	0
Suprabasin	SBSN	0	179	0	170	0	0	0	0
	IGHV1OR15-1	0	0	0	207	0	0	0	0
Attractin	ATRN	0	0	0	208	0	0	0	0
Cathepsin D;Cathepsin D light c	CTSD	0	184	0	209	0	0	0	0
Neutrophil elastase	ELANE	0	0	0	214	0	0	0	0
Angiopoietin-related protein 3	ANGPTL3	0	0	0	222	0	0	0	0
Liver-expressed antimicrobial p	LEAP2	0	0	0	226	0	0	0	0
Adipocyte enhancer-binding pro	AEBP1	0	0	0	227	0	0	0	0
Gamma-glutamylcyclotransfera	GGCT	0	182	0	230	0	0	0	0
Biotinidase	BTD	0	0	0	233	0	0	0	0
Ig lambda chain V-I region NEWM		0	0	0	239	0	0	0	0
Adiponectin	ADIPOQ	0	0	0	241	0	0	0	0
Beta-Ala-His dipeptidase	CNDP1	0	0	0	245	0	0	0	0
Complement factor H-related pr	CFHR2	0	0	0	249	0	0	0	0
Serpin B4	SERPINB4	0	0	0	250	0	0	0	0
Ig kappa chain V-I region BAN		0	0	0	254	0	0	0	0
	IGHV2-26	0	0	0	258	0	0	0	0
Complement C1r subcomponer	C1RL	0	0	0	262	0	0	0	0
Titin	TTN	0	0	0	264	0	0	0	0
Hepatocyte growth factor-like p	MST1	0	0	0	266	0	0	0	0
Eukaryotic translation initiation	EIF2S2	0	0	0	267	0	0	0	0
Putative tumor antigen NA88-A	VENTXP1	0	0	0	268	0	0	0	0

Vacuolar protein sorting-associ	VPS13A	0	0	0	269	0	0	0	0
Ig kappa chain V-II region RPM	IGKV2D-30	0	0	0	271	0	0	0	0
Alpha-synuclein	SNCA	0	0	0	272	0	0	0	0
Probable glutamate--tRNA ligas	EARS2	0	0	0	273	0	0	0	0
	IGHV1-3	0	0	0	274	0	0	0	0
	IGHV4-4	0	0	0	275	0	0	0	0
IgGFc-binding protein	FCGBP	0	0	0	276	0	0	0	0
Peptidyl-prolyl cis-trans isomera	PPIB	0	0	197	0	0	0	0	0
C-C motif chemokine 16;C-C m	CCL16	0	0	211	0	0	0	0	0
Cullin-3	CUL3	0	0	214	0	0	0	0	0
Phosphatidylinositol 5-phospha	PIP4K2A;PIP4	0	0	215	0	0	0	0	0
Myosin light polypeptide 6	MYL6	0	198	217	0	0	0	0	0
Serum deprivation-response pr	SDPR	0	195	223	0	0	0	0	0
Proprotein convertase subtilisin	PCSK6	0	0	232	0	0	0	0	0
Wiskott-Aldrich syndrome prote	WAS	0	0	236	0	0	0	0	0
Leucine-rich repeat-containing	LRRC15	0	0	239	0	0	0	0	0
Extracellular superoxide dismut	SOD3	0	0	240	0	0	0	0	0
Collagen alpha-1(XIV) chain	COL14A1	0	0	241	0	0	0	0	0
Kallikrein-7	KLK7	0	0	242	0	0	0	0	0
Gamma-glutamyl hydrolase	GGH	0	196	0	0	0	0	0	0

significant increase from 1min to 360min
significant decrease from 1min to 360min
significant increase with high flow on same material
significant increase on PMP material with same flow rate
