# Supplementary material for: Comparative study of flow rate- and material-dependent human plasma protein adsorption on oxygenator membranes and heat exchanger materials
Source: Front Cardiovasc Med. 2025 Jun 17;12:1578538. doi: 10.3389/fcvm.2025.1578538 (PMC12211862; doi:10.3389/fcvm.2025.1578538)
Supplement: Supplementary file 4 [file Table2.pdf]

Supplementary Table 2: Complete list of all measured proteins, sorted by frequency on the heparin-coated PMP membrane with a flow rate of 0.2 l/min at the indivdual time points after plasma contact (1-360min). Colored fields show significant changes (over time, between materials, or flow rates) according to the legend below.

		# of most abundance desorbed from minature devices - PMP 0.2 l/min							
**Protein names**	**Gene names**	**1 min**	**5 min**	**10 min**	**30 min**	**60 min**	**90 min**	**180 min**	**360 min**
Serum albumin	ALB	1	1	1	5	5	1	1	5
Fibrinogen alpha chain;Fibrinope	FGA	2	3	3	1	1	2	2	2
Fibrinogen beta chain;Fibrinopep	FGB	3	2	2	2	2	3	3	3
Apolipoprotein B-100;Apolipopro	APOB	4	6	6	3	4	5	5	1
Fibrinogen gamma chain	FGG	5	4	4	4	3	4	4	4
Antithrombin-III	SERPINC1	6	7	7	6	6	8	8	8
Fibronectin;Anastellin;Ugl-Y1;Ug	FN1	7	5	5	8	7	7	7	7
Apolipoprotein E	APOE	8	8	8	7	8	9	9	6
Lipopolysaccharide-binding prote	LBP	9	14	14	11	11	15	15	14
Complement C3;Complement C	C3	10	9	9	10	10	6	6	9
Ig gamma-1 chain C region	IGHG1	11	10	10	15	13	12	12	15
Complement C1q subcomponen	C1QC	12	18	18	18	27	27	27	19
Complement C1q subcomponen	C1QB	13	19	19	17	28	25	25	17
Serotransferrin	TF	14	11	11	16	18	11	11	16
Complement C1r subcomponent	C1R	15	26	26	26	44	36	36	42
Apolipoprotein A-I;Proapolipopro	APOA1	16	16	16	14	12	14	14	13
Ficolin-2	FCN2	17	17	17	9	9	10	10	12
Apolipoprotein(a)	LPA	18	34	34	13	20	16	16	10
Coagulation factor XI;Coagulatio	F11	19	24	24	22	33	37	37	29
Angiogenin	ANG	20	13	13	12	16	22	22	22
Complement C1s subcomponen	C1S	21	30	30	32	50	39	39	48
Alpha-2-macroglobulin	A2M	22	12	12	20	22	13	13	21
Ig mu chain C region	IGHM	23	21	21	19	15	19	19	20
Alpha-1-antitrypsin;Short peptide	SERPINA1	24	15	15	21	23	17	17	24
von Willebrand factor;von Willeb	VWF	25	56	56	64	72	96	96	85
Complement C1q subcomponen	C1QA	26	29	29	23	41	33	33	26
Ig kappa chain C region	IGKC	27	20	20	24	25	21	21	23
Lysozyme C	LYZ	28	33	33	27	38	42	42	41
Ig alpha-1 chain C region	IGHA1	29	23	23	29	24	26	26	32
Haptoglobin;Haptoglobin alpha c	HP	30	22	22	28	32	24	24	31
Complement C4-A;Complement	C4A	31	25	25	25	31	23	23	25
Proteoglycan 4;Proteoglycan 4 C	PRG4	32	27	27	44	76	81	81	105
Vitronectin;Vitronectin V65 subu	VTN	33	38	38	33	35	29	29	27
Immunoglobulin lambda-like poly	IGLL5;IGLC1	34	32	32	30	36	28	28	34
Complement factor H	CFH	35	40	40	39	39	31	31	38
Ig gamma-2 chain C region	IGHG2	36	28	28	38	17	30	30	35
Ribonuclease 4	RNASE4	37	43	43	42	54	60	60	54
Ig gamma-3 chain C region	IGHG3	38	35	35	36	45	32	32	39
Plasma serine protease inhibitor	SERPINA5	39	57	57	35	53	54	54	30
Hemopexin	HPX	40	31	31	40	48	34	34	50
Plasminogen;Plasmin heavy cha	PLG	41	36	36	45	43	35	35	37
Clusterin;Clusterin beta chain;Cl	CLU	42	44	44	31	30	18	18	11
Kininogen-1;Kininogen-1 heavy c	KNG1	43	45	45	58	19	46	46	71
Inter-alpha-trypsin inhibitor heav	ITIH4	44	39	39	34	40	20	20	18
Inter-alpha-trypsin inhibitor heav	ITIH2	45	42	42	48	62	40	40	36
Apolipoprotein A-IV	APOA4	46	48	48	54	49	48	48	44
Ficolin-3	FCN3	47	58	58	37	51	43	43	53
Retinoic acid receptor responder	RARRES2	48	65	65	49	58	80	80	70
Prothrombin;Activation peptide fr	F2	49	55	55	62	21	53	53	55
Vitamin D-binding protein	GC	50	41	41	59	59	44	44	72
Apolipoprotein C-I;Truncated apo	APOC1	51	62	62	47	26	69	69	43
Ceruloplasmin	CP	52	37	37	61	64	38	38	61
Apolipoprotein C-III	APOC3	53	72	72	53	65	66	66	46
Alpha-2-antiplasmin	SERPINF2	54	47	47	57	29	50	50	59
C4b-binding protein alpha chain	C4BPA	55	59	59	63	56	47	47	52
Alpha-1-antichymotrypsin;Alpha-	SERPINA3	56	46	46	70	55	45	45	66
Inter-alpha-trypsin inhibitor heav	ITIH1	57	54	54	72	98	61	61	82
Chondroadherin	CHAD	58	71	71	56	74	99	99	89
Complement factor B;Compleme	CFB	59	53	53	71	86	52	52	81
Leukocyte cell-derived chemotax	LECT2	60	60	60	50	70	93	93	161
Coagulation factor XIII A chain	F13A1	61	64	64	87	52	86	86	103
Cadherin-1;E-Cad/CTF1;E-Cad/	CDH1	62	70	70	55	96	79	79	78
Beta-2-glycoprotein 1	APOH	63	61	61	74	94	70	70	91
Plasma protease C1 inhibitor	SERPING1	64	63	63	68	14	64	64	86
Apolipoprotein D	APOD	65	87	87	69	93	82	82	60
Hepatocyte growth factor activat	HGFAC	67	133	133	99	195	124	124	0
Mannan-binding lectin serine pro	MASP1	68	84	84	51	66	55	55	73

Angiotensinogen;Angiotensin-1;/	AGT	69	52	52	75	81	63	63	74
Ig gamma-4 chain C region	IGHG4	70	50	50	79	90	51	51	69
Ribonuclease pancreatic	RNASE1	71	76	76	73	83	103	103	88
Mannan-binding lectin serine pro	MASP2	72	99	99	41	61	57	57	57
Alpha-2-HS-glycoprotein;Alpha-2	AHSG	73	51	51	78	69	62	62	76
Insulin-like growth factor-binding	IGFBP5	74	66	66	66	68	91	91	75
Lactotransferrin;Lactoferricin-H;K	LTF	75	91	91	89	119	171	171	104
Transthyretin	TTR	76	77	77	67	77	73	73	106
Protein AMBP;Alpha-1-microglob	AMBP	77	78	78	86	102	89	89	94
Insulin-like growth factor-binding	IGFBP3	78	82	82	60	67	84	84	62
Apolipoprotein A-II;Proapolipopro	APOA2	79	73	73	52	57	49	49	49
Hyaluronan-binding protein 2;Hy	HABP2	80	100	100	46	75	83	83	28
Ig heavy variable 3-72	IGHV3-72	81	75	75	81	101	74	74	87
Extracellular matrix protein 1	ECM1	82	90	90	103	111	123	123	136
Coagulation factor XIII B chain	F13B	83	79	79	130	129	117	117	139
Serum amyloid P-component;Se	APCS	84	83	83	77	87	58	58	47
Tetranectin	CLEC3B	85	110	110	92	108	112	112	100
Histidine-rich glycoprotein	HRG	86	69	69	82	106	76	76	107
Ig heavy variable 3-74; Ig heavy	IGHV3-74;IGH	87	74	74	83	103	67	67	80
Alpha-1-acid glycoprotein 1	ORM1	88	49	49	84	34	56	56	68
Coagulation factor V;Coagulation	F5	89	121	121	106	161	127	127	123
Serum paraoxonase/arylesterase	PON1	90	88	88	80	97	85	85	77
Hemoglobin subunit beta;LVV-he	HBB	91	89	89	85	60	88	88	79
Alpha-1B-glycoprotein	A1BG	92	67	67	88	85	72	72	115
Complement C5;Complement C5	C5	93	68	68	91	47	65	65	56
Heparin cofactor 2	SERPIND1	95	81	81	102	110	78	78	96
Ig kappa chain V-II region FR;Ig	IGKV2D-28	96	98	98	117	123	92	92	116
Complement factor H-related pro	CFHR1	97	93	93	94	115	94	94	67
Procollagen C-endopeptidase en	PCOLCE	98	141	141	109	127	148	148	147
Plasma kallikrein;Plasma kallikre	KLKB1	99	135	135	142	183	126	126	173
CD5 antigen-like	CD5L	100	85	85	93	99	87	87	83
Serum amyloid A2, serum amylo	SAA2-SAA4;S	101	104	104	65	71	77	77	63
Complement component C9;Cor	C9	102	86	86	112	63	68	68	51
Stromal cell-derived factor 1;SDF	CXCL12	103	92	92	76	73	90	90	65
C-C motif chemokine 18;CCL18(	CCL18	104	139	139	122	141	180	180	126
Thrombospondin-4	THBS4	106	94	94	98	131	134	134	157
Afamin	AFM	107	97	97	119	112	108	108	137
Apolipoprotein L1	APOL1	108	143	143	108	117	105	105	84
Retinol-binding protein 4;Plasma	RBP4	109	116	116	110	92	138	138	153
Ig kappa chain V-IV region	IGKV4-1	110	80	80	101	122	95	95	111
Matrix Gla protein	MGP	112	152	152	97	88	104	104	58
Joining chain of multimeric IgA a	IGJ;JCHAIN	114	114	114	95	113	106	106	101
Carboxypeptidase N catalytic ch	CPN1	115	111	111	104	124	136	136	128
Complement C4-B;Complement	C4B	116	105	105	134	125	98	98	95
Myosin-9	MYH9	117	184	184	96	136	162	162	159
Zinc-alpha-2-glycoprotein	AZGP1	118	122	122	113	137	121	121	158
Antileukoproteinase	SLPI	120	101	101	123	114	0	0	124
Apolipoprotein M	APOM	121	175	175	114	140	142	142	108
Alpha-1-acid glycoprotein 2	ORM2	122	108	108	163	155	119	119	133
Complement factor D	CFD	123	173	173	105	163	166	166	134
Coagulation factor XII;Coagulatic	F12	125	118	118	126	89	132	132	166
Hemoglobin subunit alpha	HBA1;HBA2	126	136	136	118	80	118	118	109
Ig Kappa Variable 3D-11	IGKV3D-11	127	131	131	133	0	122	122	0
Phospholipid transfer protein	PLTP	128	186	186	173	179	97	97	64
C-C motif chemokine 14;HCC-1(	CCL14	129	144	144	129	139	168	168	152
Complement component C6	C6	130	109	109	150	146	109	109	90
N-acetylmuramoyl-L-alanine ami	PGLYRP2	131	125	125	153	182	137	137	146
Glyceraldehyde-3-phosphate del	GAPDH	133	134	134	135	148	114	114	102
Complement component C8 alph	C8A	134	137	137	147	160	135	135	110
Ig heavy variable 5-51	IGHV5-51	135	126	126	164	181	147	147	168
Pleckstrin	PLEK	136	170	170	107	130	158	158	144
Haptoglobin-related protein	HPR	137	120	120	137	134	120	120	121
Fibulin-1	FBLN1	138	165	165	176	176	139	139	129
Complement component C8 beta	C8B	139	119	119	168	145	111	111	98
Carboxypeptidase N subunit 2	CPN2	140	132	132	157	144	143	143	150
Procollagen C-endopeptidase en	PCOLCE2	141	145	145	131	159	165	165	165
Properdin	CFP	143	195	195	166	79	59	59	93
Insulin-like growth factor-binding	IGFALS	144	128	128	162	180	141	141	143
Monocyte differentiation antigen	CD14	145	166	166	140	187	150	150	151

Prenylcysteine oxidase 1	PCYOX1	146	0	0	127	164	193	193	131
Apolipoprotein A-V	APOA5	147	0	0	174	184	160	160	99
Ig heavy variable 3-49	IGHV3-49	148	123	123	138	167	107	107	118
Kallistatin	SERPINA4	149	167	167	171	157	133	133	130
Apolipoprotein C-IV	APOC4	150	0	0	148	37	170	170	156
Collagen alpha-1(XVIII) chain;Er	COL18A1	151	0	0	0	192	0	0	0
Pigment epithelium-derived facto	SERPINF1	152	155	155	175	174	156	156	0
Complement component C8 gan	C8G	153	124	124	149	42	125	125	117
Tenascin-X	TNXB	154	0	0	0	0	0	0	0
Cathelicidin antimicrobial peptide	CAMP	155	146	146	156	121	159	159	141
Complement factor H-related pro	CFHR5	156	0	0	141	104	75	75	45
Dermcidin;Survival-promoting pe	DCD	157	0	0	0	214	0	0	182
Corticosteroid-binding globulin	SERPINA6	158	127	127	181	193	140	140	142
EGF-containing fibulin-like extrac	EFEMP1	159	192	192	0	211	164	164	180
Ectonucleotide pyrophosphatase	ENPP2	160	180	180	155	189	195	195	178
Zymogen granule protein 16 hon	ZG16B	161	189	189	0	0	175	175	0
Complement component C7	C7	162	129	129	144	138	115	115	92
Actin, cytoplasmic 1;Actin, cytop	ACTB	163	162	162	132	166	154	154	120
Fatty acid-binding protein, epider	FABP5	164	168	168	0	0	0	0	200
Vitamin K-dependent protein S	PROS1	165	169	169	158	152	149	149	113
Pregnancy zone protein	PZP	166	103	103	136	169	113	113	148
Neutrophil defensin 3;HP 3-56;N	DEFA3;DEFA	167	0	0	0	158	0	0	127
Inter-alpha-trypsin inhibitor heav	ITIH3	168	153	153	204	0	155	155	198
Desmocollin-3	DSC3	169	0	0	0	0	0	0	191
Complement factor I;Complemer	CFI	170	142	142	0	0	151	151	171
Alpha-enolase;Enolase	ENO1	171	0	0	0	154	0	0	0
Annexin;Annexin A2;Putative an	ANXA2;ANXA	172	160	160	205	0	0	0	0
Fructose-1,6-bisphosphatase 1	FBP1	173	0	0	194	200	0	0	0
Secreted phosphoprotein 24	SPP2	174	0	0	0	0	0	0	0
Collagen alpha-1(I) chain	COL1A1	175	0	0	0	0	0	0	0
Carboxypeptidase B2	CPB2	176	199	199	188	204	188	188	188
Desmoglein-1	DSG1	177	0	0	0	0	0	0	0
Phospholipase A2, membrane a	PLA2G2A	179	0	0	170	0	178	178	160
Selenoprotein P	SEPP1	180	196	196	185	0	0	0	187
Phosphatidylinositol-glycan-spec	GPLD1	181	193	193	0	0	184	184	199
Coagulation factor X;Factor X lig	F10	182	0	0	189	0	0	0	135
Ig delta chain C region	IGHD	183	0	0	0	191	0	0	0
Prolactin-inducible protein	PIP	184	0	0	202	0	185	185	197
Desmoplakin	DSP	186	0	0	0	0	0	0	0
Ig kappa variable 1-27	IGKV1-27	187	183	183	199	209	173	173	0
Tissue factor pathway inhibitor	TFPI	188	174	174	196	0	0	0	0
Annexin A1;Annexin	ANXA1	189	0	0	0	0	0	0	0
Tartrate-resistant acid phosphata	ACP5	190	0	0	0	0	0	0	0
Leucine-rich alpha-2-glycoprotei	LRG1	0	150	150	190	0	161	161	0
Lumican	LUM	0	185	185	187	205	181	181	0
Complement C2;Complement C2	C2	0	0	0	0	82	0	0	0
Thyroxine-binding globulin	SERPINA7	0	171	171	184	0	177	177	0
Serum amyloid A-1 protein;Amyl	SAA1	0	0	0	201	0	0	0	0
Galectin-3-binding protein	LGALS3BP	0	200	200	0	0	189	189	202
Serum amyloid A-2 protein	SAA2	0	0	0	0	203	0	0	163
Glutathione peroxidase 3	GPX3	0	191	191	186	188	144	144	97
Phosphatidylcholine-sterol acyltr	LCAT	0	0	0	0	0	191	191	181
C-reactive protein;C-reactive pro	CRP	0	0	0	0	0	152	152	132
Talin-1	TLN1	0	0	0	0	0	0	0	194
Sulfhydryl oxidase 1	QSOX1	0	0	0	0	0	187	187	0
Polymeric immunoglobulin recep	PIGR	0	0	0	0	213	0	0	0
Transforming growth factor-beta-	TGFBI	0	0	0	0	0	0	0	0
Protein S100-A9	S100A9	0	163	163	182	0	0	0	0
C4b-binding protein beta chain	C4BPB	0	0	0	198	212	0	0	195
Cathepsin D;Cathepsin D light cl	CTSD	0	188	188	0	0	0	0	0
Protein S100-A8;Protein S100-A	S100A8	0	140	140	178	0	0	0	175
Fermitin family homolog 3	FERMT3	0	0	0	0	0	0	0	201
Protein disulfide-isomerase A3	PDIA3	0	0	0	0	0	0	0	0
Cholesteryl ester transfer protein	CETP	0	0	0	100	95	41	41	33
Multimerin-1;Platelet glycoprotei	MMRN1	0	0	0	200	208	0	0	0
Peroxiredoxin-6	PRDX6	0	0	0	0	0	186	186	0
Band 3 anion transport protein	SLC4A1	0	0	0	0	177	0	0	0
Adipocyte plasma membrane-as	APMAP	0	0	0	0	109	0	0	0
CD9 antigen	CD9	0	0	0	0	91	0	0	0

Angiopoietin-related protein 3	ANGPTL3	0	181	181	0	0	0	0	0
Histone H4	HIST1H4A	0	177	177	152	162	163	163	114
Myeloperoxidase;Myeloperoxida	MPO	0	198	198	0	0	0	0	0
Alcohol dehydrogenase 4	ADH4	0	0	0	0	0	0	0	0
DnaJ homolog subfamily C mem	DNAJC3	0	0	0	193	199	0	0	0
GTP-binding nuclear protein Rar	RAN	0	194	194	0	0	190	190	196
Bone morphogenetic protein 1	BMP1	0	0	0	0	0	0	0	0
Tsukushin	TSKU	0	0	0	0	0	0	0	170
BPI fold-containing family A men	BPIFA1	0	0	0	0	0	0	0	203
Lipoprotein lipase	LPL	0	190	190	192	202	169	169	176
Proprotein convertase subtilisin/l	PCSK6	0	0	0	0	175	0	0	0
Ig heavy variable 3/OR16-9	IGHV3OR16-	0	115	115	0	186	130	130	154
Ig kappa chain V-I region HK102	IGKV1-5	0	0	0	0	0	0	0	179
Ig Lambda Variable 8-61	IGLV8-61	0	0	0	180	0	0	0	174
Cofilin-1	CFL1	0	0	0	0	84	0	0	0
Adenylyl cyclase-associated pro	CAP1	0	0	0	0	210	0	0	0
Semaphorin 3B	SEMA3B	0	0	0	0	0	0	0	206
Phospholipase A1 member A	PLA1A	0	0	0	0	0	0	0	0
Ig heavy chain variable region 1-	IGHV1-18	0	147	147	0	0	128	128	0
Protein S100-A7;Protein S100-A	S100A7;S100	0	161	161	191	0	0	0	184
Protein phosphatase 1 regulator	PPP1R10	0	0	0	0	0	172	172	0
Ig heavy variable 1-69-2	IGHV1-69-2	0	0	0	179	0	0	0	0
Ig heavy variable 6-1	IGHV6-1	0	0	0	0	0	153	153	0
Sex hormone-binding globulin	SHBG	0	0	0	0	0	0	0	0
Prostaglandin-H2 D-isomerase	PTGDS	0	0	0	0	0	0	0	0
Insulin-like growth factor-binding	IGFBP4	0	0	0	0	116	0	0	0
Insulin-like growth factor I	IGF1	0	0	0	0	0	0	0	0
Platelet-activating factor acetylhy	PLA2G7	0	0	0	0	168	0	0	0
Peptidyl-prolyl cis-trans isomera	PPIB	0	0	0	0	0	0	0	0
Caldesmon	CALD1	0	0	0	177	0	0	0	0
Suprabasin	SBSN	0	182	182	0	197	0	0	0
Cystatin-A;Cystatin-A, N-termina	CSTA	0	138	138	0	0	0	0	177
Arginase-1	ARG1	0	157	157	0	0	0	0	0
Protein-glutamine gamma-glutar	TGM3	0	178	178	0	143	0	0	0
Serpin B3	SERPINB3	0	159	159	0	0	194	194	0
Protein disulfide-isomerase A5	PDIA5	0	0	0	203	215	0	0	0
Adipocyte enhancer-binding prot	AEBP1	0	179	179	0	0	0	0	0
Caspase-14;Caspase-14 subuni	CASP14	0	197	197	0	0	0	0	0
Semenogelin-1;Alpha-inhibin-92	SEMG1	0	113	113	154	170	167	167	155
Olfactomedin-like protein 3	OLFML3	0	172	172	0	0	0	0	0
Liver-expressed antimicrobial pe	LEAP2	0	0	0	0	78	0	0	0
Kallikrein-7	KLK7	0	0	0	0	128	0	0	0
Semenogelin-2	SEMG2	0	149	149	0	0	0	0	0
Laminin subunit alpha-4	LAMA4	0	0	0	0	0	0	0	0
Signal peptide, CUB and EGF-lik	SCUBE2	0	164	164	197	0	183	183	0
Asporin	ASPN	0	187	187	0	206	192	192	0
Ras-related protein Rap-1b;Ras-	RAP1B;RAP1	0	0	0	0	100	0	0	0
Nephronectin	NPNT	0	156	156	145	151	176	176	164
Platelet factor 4;Platelet factor 4,	PF4;PF4V1	0	130	130	165	156	0	0	149
Bactericidal permeability-increas	BPI	0	0	0	183	190	0	0	190
Deoxyribonuclease gamma	DNASE1L3	0	0	0	167	207	0	0	192
Eosinophil cationic protein	RNASE3	0	0	0	161	185	0	0	167
Complement C1q tumor necrosis	C1QTNF3-AM	0	0	0	0	0	0	0	0
Myosin Light Chain 6	MYL6	0	0	0	0	201	0	0	0
aminopeptidase puromycin sens	NPEPPS	0	0	0	0	153	0	0	0

(orange)	significant increase from 1min to 360min
(blue)	significant decrease from 1min to 360min
(green)	significant increase with low flow on same material
(grey)	significant increase on PMP material with same flow rate
