# Supplementary material for: Comparative study of flow rate- and material-dependent human plasma protein adsorption on oxygenator membranes and heat exchanger materials
Source: Front Cardiovasc Med. 2025 Jun 17;12:1578538. doi: 10.3389/fcvm.2025.1578538 (PMC12211862; doi:10.3389/fcvm.2025.1578538)
Supplement: Supplementary file 5 [file Table3.pdf]

Supplementary Table 3: Complete list of all measured proteins, sorted by frequency on the heparin-coated PET membrane with a flow rate of 1 l/min at the indivdual time points after plasma contact (1-360min). Colored fields show significant changes (over time, between materials, or flow rates) according to the legend below.

		# of most abundance desorbed from minature devices - PET 1 l/min							
**Protein names**	**Gene names**	**1 min**	**5 min**	**10 min**	**30 min**	**60 min**	**90 min**	**180 min**	**360 min**
Serum albumin	ALB	4	4	2	3	3	3	5	1
Apolipoprotein B-100;Apolipopro	APOB	5	6	5	5	5	5	2	2
Fibrinogen alpha chain;Fibrinope	FGA	1	1	1	1	1	1	1	3
Fibrinogen beta chain;Fibrinope	FGB	2	2	3	2	2	2	3	4
Fibrinogen gamma chain	FGG	3	3	4	4	4	4	4	5
Inter-alpha-trypsin inhibitor heav	ITIH4	39	22	17	10	9	8	6	6
Complement C3;Complement C	C3	10	9	9	9	8	6	8	7
Fibronectin;Anastellin;Ugl-Y1;Ug	FN1	7	5	7	6	7	7	7	8
Clusterin;Clusterin beta chain;Cl	CLU	33	34	28	18	19	13	12	9
Ig gamma-1 chain C region	IGHG1	16	11	11	11	12	12	13	10
Apolipoprotein E	APOE	8	8	8	7	10	10	10	11
Antithrombin-III	SERPINC1	6	7	6	8	6	9	9	12
Lipopolysaccharide-binding prot	LBP	9	13	10	12	11	11	11	13
Complement C4-A;Complement	C4A	32	26	25	19	17	17	14	14
Serotransferrin	TF	14	12	13	13	15	15	19	15
Apolipoprotein(a)	LPA	23	24	24	16	14	14	15	16
Vitronectin;Vitronectin V65 subu	VTN	34	30	32	26	26	25	20	17
Alpha-2-macroglobulin	A2M	19	16	16	17	20	18	17	18
Apolipoprotein A-I;Proapolipopro	APOA1	15	15	14	14	18	19	18	19
Ficolin-2	FCN2	18	14	15	15	13	16	16	20
Alpha-1-antitrypsin;Short peptide	SERPINA1	21	17	19	23	25	24	24	21
Hyaluronan-binding protein 2;Hy	HABP2	81	84	60	33	38	20	23	22
Ig kappa chain C region	IGKC	29	20	22	24	24	28	27	23
Complement C1q subcomponen	C1QB	13	21	18	22	22	26	22	24
Ig mu chain C region	IGHM	22	18	21	21	21	23	28	25
Plasma serine protease inhibitor	SERPINA5	47	49	48	30	35	22	21	26
Inter-alpha-trypsin inhibitor heav	ITIH2	50	45	39	36	37	35	31	27
Complement C1q subcomponen	C1QC	11	19	20	25	23	27	25	28
Angiogenin	ANG	12	10	12	20	16	21	26	29
Haptoglobin;Haptoglobin alpha c	HP	27	25	23	28	30	29	32	30
Ig alpha-1 chain C region	IGHA1	31	27	30	27	29	30	29	31
Ig gamma-3 chain C region	IGHG3	40	38	35	31	31	31	30	32
Plasminogen;Plasmin heavy cha	PLG	45	35	41	38	36	37	36	33
Serum amyloid P-component;Se	APCS	76	73	76	72	63	45	42	34
Immunoglobulin lambda-like poly	IGLL5;IGLC1	37	32	33	32	34	36	33	35
Prothrombin;Activation peptide f	F2	68	54	50	45	43	41	37	36
Apolipoprotein A-IV	APOA4	43	47	42	46	45	46	43	37
Alpha-2-antiplasmin	SERPINF2	62	42	47	40	41	40	35	38
Complement C1q subcomponen	C1QA	20	33	29	34	28	33	34	39
Ig gamma-2 chain C region	IGHG2	44	37	36	41	39	44	40	40
Cholesteryl ester transfer protein	CETP	137	126	107	50	50	38	39	41
Hemopexin	HPX	38	39	37	43	44	42	49	42
Complement component C9;Co	C9	92	89	94	62	48	43	46	43
Kininogen-1;Kininogen-1 heavy	KNG1	46	44	43	48	46	52	48	44
Inter-alpha-trypsin inhibitor heav	ITIH1	65	67	57	58	59	55	52	45
Ig gamma-4 chain C region	IGHG4	75	66	73	65	73	65	60	46
Alpha-1-antichymotrypsin;Alpha-	SERPINA3	63	59	51	54	58	56	57	47
Complement C5;Complement C	C5	88	78	85	59	53	50	50	48
Inter-alpha-trypsin inhibitor heav	ITIH4	0	0	0	113	0	87	44	49
Coagulation factor XI;Coagulatio	F11	17	29	31	29	27	32	38	50
Complement factor B;Compleme	CFB	59	58	55	55	52	51	51	51
Complement factor H	CFH	41	40	40	39	32	34	47	52
Ceruloplasmin	CP	55	48	46	49	57	49	56	53
Apolipoprotein L1	APOL1	111	117	102	88	94	71	59	54
von Willebrand factor;von Willeb	VWF	77	28	59	37	51	47	45	55
Alpha-2-HS-glycoprotein;Alpha-2	AHSG	69	63	64	63	60	61	61	56
C4b-binding protein alpha chain	C4BPA	54	56	54	53	49	58	55	57
Complement C1r subcomponen	C1R	25	36	27	35	33	48	53	58
Heparin cofactor 2	SERPIND1	87	87	74	68	62	63	58	59
Angiotensinogen;Angiotensin-1;A	AGT	67	51	53	57	61	59	54	60
Vitamin D-binding protein	GC	57	50	44	52	64	60	65	61
Coagulation factor V;Coagulatio	F5	95	102	87	79	86	90	62	62
Actin, cytoplasmic 1;Actin, cytop	ACTB	91	82	108	90	89	74	41	63
Glutathione peroxidase;Glutathi	GPX3	0	0	160	149	134	112	79	64
Complement factor H-related pro	CFHR5	0	0	159	135	79	53	68	65
Plasma protease C1 inhibitor	SERPING1	71	75	75	74	85	77	74	66
Complement C1s subcomponen	C1S	28	43	34	42	40	57	64	67

Alpha-1-acid glycoprotein 1	ORM1	102	60	66	64	77	64	102	68
Fibulin-1	FBLN1	0	118	155	101	105	93	63	69
Phospholipid transfer protein	PLTP	123	0	165	103	97	73	67	70
Tsukushin	TSKU	0	0	0	174	149	108	75	71
Protein AMBP;Alpha-1-microglo	AMBP	83	70	84	81	82	76	77	72
Serum paraoxonase/arylesteras	PON1	94	91	83	76	78	72	72	73
Complement component C7	C7	131	135	135	106	99	82	85	74
EGF-containing fibulin-like extra	EFEMP1	213	0	153	110	101	107	89	75
Apolipoprotein A-II;Proapolipopr	APOA2	53	74	58	56	76	70	73	76
Apolipoprotein D	APOD	70	88	82	78	71	69	70	77
Proteoglycan 4;Proteoglycan 4 (	PRG4	26	23	26	47	54	75	76	78
Complement component C8 bet	C8B	124	128	126	119	100	91	84	79
Apolipoprotein C-I;Truncated ap	APOC1	42	61	56	70	75	68	90	80
Beta-2-glycoprotein 1	APOH	66	71	67	97	91	84	109	81
Serum amyloid A-4 protein	SAA2-SAA4;S/	97	90	93	93	83	92	95	82
Vitamin K-dependent protein S	PROS1	145	144	140	136	141	129	113	83
Lysozyme C;Lysozyme	LYZ	30	31	38	44	42	54	78	84
Inter-alpha-trypsin inhibitor heav	ITIH3	166	150	170	155	144	114	126	85
Alpha-1B-glycoprotein	A1BG	86	85	88	85	95	85	105	86
Ig heavy chain V-III region BUT	IGHV3-74;IGH	79	86	89	82	103	94	98	87
Kallistatin	SERPINA4	132	127	130	123	120	111	106	88
Ig heavy variable 3-72	IGHV3-72	101	80	80	92	87	79	86	89
Hemoglobin subunit beta;LVV-h	HBB	24	64	49	51	88	86	103	90
Transthyretin	TTR	78	81	72	77	90	96	92	91
Complement C4-B;Complement	C4B	122	110	103	98	93	80	94	92
Ribonuclease 4	RNASE4	35	41	45	61	47	62	71	93
Ficolin-3	FCN3	56	55	63	66	65	83	87	94
Complement component C6	C6	169	134	124	115	102	95	101	95
Histidine-rich glycoprotein	HRG	74	65	77	91	80	98	104	96
Apolipoprotein C-III	APOC3	51	72	71	60	84	81	93	97
Mannan-binding lectin serine pro	MASP2	96	77	81	75	56	67	91	98
Insulin-like growth factor-binding	IGFBP3	61	76	68	86	69	78	88	99
Apolipoprotein C-II;Proapolipopr	APOC4-APOC	72	94	86	67	68	66	80	100
Ig kappa chain V-I region AU;Ig	kappa chain V-I	112	116	100	104	108	110	138	101
Coagulation factor XIII A chain	F13A1	73	52	91	80	96	100	97	102
Ig kappa chain V-III region B6	IGKV3-20	130	103	90	94	132	0	107	103
Ig kappa chain V-II region FR	IGKV2D-28	98	105	92	105	122	106	112	104
Complement component C8 alpl	C8A	135	139	142	147	113	109	117	105
Ig kappa chain V-IV region	IGKV4-1	115	106	96	108	131	134	131	106
Complement component C8 gar	C8G	128	124	125	125	121	113	120	107
Pregnancy zone protein	PZP	223	143	98	111	123	125	83	108
CD5 antigen-like	CD5L	104	96	99	96	98	102	118	109
N-acetylmuramoyl-L-alanine am	PGLYRP2	129	138	144	118	128	118	127	110
C-reactive protein;C-reactive pro	CRP	200	0	0	176	177	177	161	111
Ribonuclease pancreatic	RNASE1	64	53	65	102	74	97	124	112
Insulin-like growth factor-binding	IGFALS	149	122	141	141	139	122	133	113
Band 3 anion transport protein	SLC4A1	209	158	194	128	110	103	123	114
Corticosteroid-binding globulin	SERPINA6	160	142	134	144	147	136	146	115
Coagulation factor X;Factor X lig	F10	195	0	173	157	168	150	154	116
Haptoglobin-related protein	HPR	153	119	119	114	115	116	125	117
Carboxypeptidase N subunit 2	CPN2	167	112	137	140	137	135	136	118
Integrin alpha-IIb;Integrin alpha-	ITGA2B	0	0	0	213	174	164	152	119
Leukocyte cell-derived chemota:	LECT2	48	46	52	71	70	89	100	120
Complement factor H-related pro	CFHR1	103	101	115	116	112	105	115	121
Alpha-1-antitrypsin;Short peptide	SERPINA1	99	111	111	100	143	123	195	122
Apolipoprotein A-V	APOA5	138	0	167	161	124	126	144	123
Cadherin-1;E-Cad/CTF1;E-Cad/	CDH1	49	57	61	69	66	101	108	124
Ig lambda chain V-III region SH	IGLV3-19	148	0	120	117	142	0	135	125
Flavin reductase (NADPH)	BLVRB	183	0	0	0	0	200	197	126
Alpha-1-acid glycoprotein 2	ORM2	0	98	127	129	140	128	171	127
Retinoic acid receptor responde	RARRES2	52	69	70	83	67	88	111	128
Hemoglobin subunit alpha	HBA1;HBA2	36	95	79	89	119	120	149	129
Chondroadherin	CHAD	60	62	62	84	81	99	116	130
Ig kappa chain V-III region VG	IGKV3D-11	0	0	129	139	0	166	129	131
Afamin	AFM	106	123	123	121	127	121	155	132
Apolipoprotein M	APOM	150	152	132	130	126	124	143	133
Glyceraldehyde-3-phosphate de	GAPDH	107	114	117	127	114	115	110	134
Mannan-binding lectin serine pro	MASP1	85	99	95	87	72	104	121	135
Carboxypeptidase N catalytic ch	CPN1	118	100	106	122	125	130	139	136

Transforming growth factor-beta	TGFBI	221	0	178	168	165	148	132	137
Fibronectin;Anastellin;Ugl-Y1;Ug	FN1	136	115	0	131	0	0	163	138
Retinol-binding protein 4;Plasma	RBP4	119	129	112	146	135	140	167	139
Plasma kallikrein;Plasma kallikre	KLKB1	120	140	136	150	157	153	166	140
Procollagen C-endopeptidase er	PCOLCE	125	153	133	154	145	151	176	141
Immunoglobulin J chain	IGJ;JCHAIN	108	97	104	109	118	119	142	142
Histone H4	HIST1H4A	162	0	169	160	166	158	122	143
Matrix Gla protein	MGP	175	0	147	126	104	117	157	144
Prenylcysteine oxidase 1	PCYOX1	193	0	177	162	155	149	187	145
Integrin beta;Integrin beta-3	ITGB3	0	0	0	0	196	0	282	146
Pigment epithelium-derived facto	SERPINF1	155	146	154	163	170	168	220	147
Monocyte differentiation antigen	CD14	141	0	151	142	130	133	159	148
Ig heavy chain V-III region CAM	IGHV3-23	0	0	118	133	0	145	147	149
Coagulation factor XII;Coagulati	F12	147	125	146	152	154	147	160	150
Zinc-alpha-2-glycoprotein	AZGP1	110	137	122	151	156	154	202	151
Fermitin family homolog 3	FERMT3	0	0	0	0	180	182	185	152
Properdin	CFP	146	156	162	73	55	39	114	153
Ig heavy variable 3-15	IGHV3-15	0	0	138	112	133	142	173	154
Collagen alpha-3(VI) chain	COL6A3	0	0	0	0	0	172	198	155
Actin, alpha skeletal muscle;Acti	ACTA1;ACTC1	0	0	0	0	0	0	151	156
Tetranectin	CLEC3B	100	109	116	138	117	138	164	157
Ig heavy variable 5-51	IGHV5-51	157	133	156	158	160	161	181	158
Thrombospondin-4	THBS4	90	93	101	107	116	132	188	159
Sex hormone-binding globulin	SHBG	230	0	182	202	0	163	206	160
Peptidyl-glycine alpha-amidating	PAM	0	0	0	194	185	197	193	161
Stromal cell-derived factor 1;SD	CXCL12	84	92	78	95	138	139	134	162
Protein Z-dependent protease in	SERPINA10	207	0	191	203	0	175	251	163
Peroxiredoxin-6	PRDX6	238	0	0	212	207	190	216	164
Fibrinogen-like protein 1	FGL1	0	0	0	0	0	0	233	165
Ig heavy variable 3OR1-16	IGHV3OR16-9	117	0	121	166	0	0	192	166
Thyroxine-binding globulin	SERPINA7	203	157	183	199	0	196	0	167
Coagulation factor XIII B chain	F13B	109	79	150	137	163	152	180	168
Extracellular matrix protein 1	ECM1	93	83	114	132	129	160	182	169
Ig lambda chain V-I region HA	IGLV1-44	182	0	168	175	171	188	175	170
Coagulation factor VII;Factor VII	F7	0	0	203	206	0	187	201	171
Phosphatidylcholine-sterol acyltr	LCAT	0	0	0	0	0	191	224	172
Ig lambda chain V-IV region Hil;Ig lambda chain		0	0	110	153	0	155	148	173
Insulin-like growth factor-binding	IGFBP5	58	68	69	99	92	127	150	174
Kininogen-1;Kininogen-1 heavy	KNG1	0	0	0	0	0	0	0	175
Carboxypeptidase B2	CPB2	180	0	186	185	0	176	230	176
Sulfhydryl oxidase 1	QSOX1	206	0	204	210	198	199	252	177
Ig heavy variable 3-49	IGHV3-49	133	145	148	148	151	144	156	178
Apolipoprotein C-IV	APOC4	0	0	158	165	146	137	165	179
Phosphatidylinositol-glycan-spec	GPLD1	202	0	0	215	201	186	211	180
Fetuin-B	FETUB	219	0	0	200	193	0	266	181
Lipoprotein lipase	LPL	192	151	176	180	175	198	213	182
Coagulation factor IX;Coagulatio	F9	197	0	180	198	192	183	240	183
Talin-1	TLN1	226	0	207	0	205	203	219	184
Serum amyloid A-2 protein	SAA2	0	0	0	186	0	0	0	185
Ig delta chain C region	IGHD	0	0	0	188	172	179	215	186
Neutrophil defensin 3;HP 3-56;N	DEFA3;DEFA1	134	0	175	182	191	0	0	187
Secreted phosphoprotein 24	SPP2	0	0	188	0	0	0	221	188
Ig kappa chain V-I region Daudi;	IGKV1-6;IGKV	0	0	0	178	0	0	277	189
Ig kappa variable 1-27	IGKV1-27	0	0	0	179	0	0	261	190
Ig light chain variable 8-61	IGLV8-61	0	0	164	0	182	0	212	191
Complement factor I;Compleme	CFI	165	147	174	177	195	178	0	192
Pyruvate kinase PKM;Pyruvate k	PKM	220	0	0	0	0	0	145	193
78 kDa glucose-regulated protei	HSPA5	0	0	0	0	0	0	205	194
Myosin-9	MYH9	126	131	109	134	107	141	178	195
Leucine-rich alpha-2-glycoprotei	LRG1	164	0	171	171	203	180	0	196
Lumican	LUM	172	0	0	189	0	171	253	197
Lactotransferrin;Lactoferricin-H;L	LTF	80	136	105	124	111	143	265	198
Ig lambda chain V-I region NEW	IGLV1-51	0	0	0	0	0	0	0	199
Alpha-1,3-mannosyl-glycoprotein	MGAT1	0	0	0	0	0	195	214	200
Histone H2B type 1-L;Histone H	HIST1H2BL;HI	184	0	128	173	150	162	140	201
Complement factor D	CFD	159	149	152	169	152	156	210	202
Inter-alpha-trypsin inhibitor heav	ITIH4	0	0	0	0	204	205	241	203
Histone H3;Histone H3.3C;Histo	HIST2H3PS2;H	188	0	0	0	189	0	162	204
Spectrin beta chain, erythrocytic	SPTB	0	0	0	0	0	0	288	205

C4b-binding protein beta chain	C4BPB	0	0	0	211	197	0	0	206
Nephronectin	NPNT	163	113	143	159	148	157	244	207
Ankyrin-1	ANK1	0	0	0	0	199	181	235	208
Procollagen C-endopeptidase er	PCOLCE2	116	0	157	172	162	169	200	209
14-3-3 protein zeta/delta	YWHAZ	0	0	0	0	0	0	174	210
Ig kappa chain V-I region HK102	IGKV1-5	0	0	0	0	0	189	226	211
Ig heavy chain V-I region HG3	IGJKV1-5	0	0	0	191	194	0	263	212
Erythrocyte band 7 integral mem	STOM	0	0	0	0	0	0	0	213
Serum amyloid A-1 protein;Amy	SAA1	0	0	0	195	176	202	258	214
Pleckstrin	PLEK	127	130	161	156	136	165	243	215
Selenoprotein P	SEPP1	174	0	172	184	187	201	0	216
Hepatocyte growth factor activat	HGFAC	168	0	199	187	173	194	0	217
Mannosyl-oligosaccharide 1,2-a	MAN1A1	0	0	0	0	0	0	0	218
Phospholipase A2, membrane a	PLA2G2A	216	0	193	170	169	173	0	219
Multimerin-2	MMRN2	0	0	0	0	0	0	301	220
Hyaluronidase-1	HYAL1	0	0	0	0	0	0	0	221
Vitamin K-dependent protein C;V	PROC	0	0	0	0	0	0	278	222
14-3-3 protein sigma	SFN	0	0	0	0	0	0	96	223
GTP-binding nuclear protein Ran	RAN	0	0	187	214	178	192	256	224
Endoplasmin	HSP90B1	0	0	0	0	0	0	0	225
Ig heavy chain V-III region DOB	IGHV3-9	0	0	0	0	0	0	0	226
Spectrin alpha chain, erythrocyti	SPTA1	0	0	0	0	0	0	0	227
Cathelicidin antimicrobial peptide	CAMP	154	121	149	181	161	167	0	228
Erythrocyte membrane protein b	EPB42	0	0	0	0	0	0	0	229
Tryptophan 5-hydroxylase 1	TPH1	0	0	0	0	0	0	0	230
N-acetylglucosamine-1-phospho	GNPTG	0	0	0	0	0	0	0	231
ADAMTS-like protein 4	ADAMTSL4	0	0	0	0	0	0	0	232
Galectin-3-binding protein	LGALS3BP	228	0	0	216	0	185	242	233
Tenascin-X	TNXB	142	0	0	0	0	0	0	234
Basement membrane-specific he	HSPG2	0	0	0	0	0	0	0	235
Inhibin beta C chain	INHBC	0	0	0	0	0	0	0	236
Integrin-linked protein kinase	ILK	0	0	0	0	0	0	0	237
Profilin-1	PFN1	0	0	0	0	0	0	269	238
Complement factor H-related pro	CFHR4	0	0	0	0	0	0	0	239
Anthrax toxin receptor 2	ANTXR2	0	0	0	0	0	0	0	240
Solute carrier family 2, facilitated	SLC2A1	0	0	0	0	0	0	0	241
Serine protease HTRA1	HTRA1	0	0	0	0	0	0	294	242
Vinculin	VCL	0	0	0	0	0	0	0	243
Tryptophan--tRNA ligase, cytopl	WARS	0	0	0	0	0	0	284	244
Macrophage receptor MARCO	MARCO	0	0	0	0	0	0	0	245
Tenascin	TNC	0	0	0	0	0	0	0	246
Protein ERGIC-53	LMAN1	0	0	0	0	0	0	0	247
Cartilage acidic protein 1	CRTAC1	0	0	0	0	0	0	0	248
Inhibin beta E chain	INHBE	0	0	0	0	0	0	0	249
Pyruvate kinase PKM;Pyruvate k	PKM	0	0	0	0	0	0	0	250
Serpin B3	SERPINB3	0	0	0	0	0	0	66	0
Protein S100-A9	S100A9	204	0	200	217	0	0	69	0
Protein S100-A8;Protein S100-A	S100A8	0	0	0	0	0	0	81	0
Galectin-7	LGALS7	0	132	0	0	0	0	82	0
Desmoplakin	DSP	89	0	0	208	0	0	99	0
Heat shock protein beta-1	HSPB1	0	0	0	0	0	0	119	0
Calmodulin-like protein 5	CALML5	0	0	0	0	0	0	128	0
Serpin B4	SERPINB4	0	0	0	0	0	0	130	0
Ig heavy chain V-II region NEWM	IGHV4-61	0	120	131	120	106	131	137	0
Histone H2A type 1-J;Histone H	HIST1H2AJ;HI	178	0	113	145	153	204	141	0
Elongation factor 1-alpha 1;Puta	EEF1A1;EEF1A	0	0	202	201	0	210	153	0
Suprabasin	SBSN	0	0	0	0	0	0	158	0
Epiplakin	EPPK1	0	0	0	0	0	0	168	0
Fructose-bisphosphate aldolase	ALDOA	0	0	0	0	0	0	169	0
Ig lambda chain V-III region LOI	IGLV3 LOI	0	0	0	0	0	0	170	0
Fatty acid-binding protein, epide	FABP5	156	0	201	0	0	0	172	0
Polyubiquitin-C;Ubiquitin;Ubiquit	UBC;UBB;RPS	144	0	0	0	0	0	177	0
Heat shock 70 kDa protein 1B;H	HSPA1B;HSPA	0	0	0	0	0	0	179	0
Tubulin alpha-3E chain	TUBA3E	0	0	0	0	0	0	183	0
Annexin A2;Annexin;Putative an	ANXA2;ANXA2	176	0	0	218	0	0	184	0
Junction plakoglobin	JUP	105	0	0	204	0	0	186	0
Heat shock protein HSP 90-beta	HSP90AB1	0	0	0	0	0	0	189	0
Alpha-enolase;Enolase	ENO1	170	0	0	0	0	0	190	0
Glutamine synthetase	GLUL	0	0	0	0	0	0	191	0

Involucrin	IVL	0	0	0	0	0	0	194	0
Protein S100-A11;Protein S100-	S100A11	0	0	0	0	0	0	196	0
Calmodulin-1; Calmodulin-2; Ca	CALM2;CALM3	0	0	0	0	0	0	199	0
Calmodulin-like protein 3	CALML3	0	0	0	0	0	0	203	0
Protein-glutamine gamma-glutar	TGM3	186	0	0	0	0	0	204	0
F-box only protein 50	NCCRP1	0	0	0	0	0	0	207	0
Triosephosphate isomerase	TPI1	235	0	0	0	0	0	208	0
Peptidyl-prolyl cis-trans isomera	PPIA	0	0	0	0	0	0	209	0
Eukaryotic initiation factor 4A-I	EIF4A1	0	0	0	0	0	0	217	0
Protein S100-A7;Protein S100-A	S100A7;S100A	218	0	0	0	0	0	218	0
Thymidine phosphorylase	TYMP	0	0	0	0	0	0	222	0
Serpin B5	SERPINB5	0	0	0	0	0	0	223	0
Tubulin beta-4B chain;Tubulin b	TUBB4B;TUBB	0	0	0	0	0	0	225	0
Cathepsin D;Cathepsin D light c	CTSD	0	0	0	0	0	0	227	0
Protein disulfide-isomerase	P4HB	0	0	0	0	0	0	228	0
Prelamin-A/C;Lamin-A/C	LMNA	0	0	0	0	0	0	229	0
Phosphoglycerate kinase 1	PGK1	234	0	0	0	0	0	231	0
Transitional endoplasmic reticulu	VCP	0	0	0	0	0	0	232	0
Desmoglein-1	DSG1	113	0	0	192	0	0	234	0
Peroxiredoxin-1	PRDX1	191	0	0	0	0	0	236	0
L-lactate dehydrogenase A chai	LDHA	0	0	0	0	0	0	237	0
Thioredoxin	TXN	0	0	0	0	0	0	238	0
Plakophilin-1	PKP1	198	0	0	0	0	0	239	0
Glutathione S-transferase P	GSTP1	0	0	0	0	0	0	245	0
Protein POF1B	POF1B	0	0	0	0	0	0	246	0
Cellular retinoic acid-binding pro	CRABP2	0	0	0	0	0	0	247	0
Cystatin-B	CSTB	0	0	0	0	0	0	248	0
Cofilin-1	CFL1	0	0	0	0	0	0	249	0
Heat shock cognate 71 kDa prot	HSPA8	0	0	0	0	0	0	250	0
60S acidic ribosomal protein P2	RPLP2	0	0	0	0	0	0	254	0
Periplakin	PPL	0	0	0	0	0	0	255	0
Neuroblast differentiation-associ	AHNAK	0	0	0	0	0	0	257	0
Tubulin alpha-4A chain	TUBA4A	0	0	0	0	0	0	259	0
C-C motif chemokine 18;CCL18	CCL18	139	148	163	164	159	159	260	0
Insulin-degrading enzyme	IDE	0	0	0	0	0	0	262	0
14-3-3 protein epsilon	YWHAE	0	0	0	0	0	0	264	0
Ezrin;Radixin	EZR;RDX	0	0	0	0	0	0	267	0
Gamma-glutamylcyclotransferas	GGCT	0	0	0	0	0	0	268	0
40S ribosomal protein SA	RPSA	0	0	0	0	0	0	270	0
Heat shock protein HSP 90-alph	HSP90AA1	0	0	0	0	0	0	271	0
Platelet factor 4;Platelet factor 4	PF4;PF4V1	151	107	139	196	167	174	272	0
Ectonucleotide pyrophosphatase	ENPP2	208	0	0	193	158	184	273	0
Ig kappa chain variable 3D-15	IGKV3D-15	0	0	0	0	0	0	274	0
Kallikrein-6	KLK6	0	0	0	0	0	0	275	0
60S acidic ribosomal protein P1	RPLP1	0	0	0	0	0	0	276	0
Tubulin alpha-1B chain;Tubulin a	TUBA1B;TUBA	0	0	0	0	0	0	279	0
Elongation factor 2	EEF2	0	0	0	0	0	0	280	0
Caspase-14;Caspase-14 subuni	CASP14	236	0	206	0	0	0	281	0
Alpha-actinin-1;Alpha-actinin-4;A	ACTN1;ACTN4	0	0	0	0	0	0	283	0
Complement C2;Complement C	C2	0	0	0	0	0	209	285	0
Retroviral-like aspartic protease	ASPRV1	0	0	0	0	0	0	286	0
Calreticulin	CALR	0	0	0	0	0	0	287	0
Gasdermin-A	GSDMA	0	0	0	0	0	0	289	0
C-C motif chemokine 14;HCC-1(	CCL14	121	108	145	167	164	0	290	0
60S acidic ribosomal protein P0;	RPLP0;RPLP0	0	0	0	0	0	0	291	0
Protein disulfide-isomerase A3	PDIA3	0	0	0	0	0	0	292	0
Adenylyl cyclase-associated pro	CAP1	0	0	0	0	0	0	293	0
Malate dehydrogenase, cytoplas	MDH1	0	0	0	0	0	0	295	0
Actin, cytoplasmic 2;Actin, cytop	ACTG1	0	0	0	0	0	0	296	0
Glucose-6-phosphate isomerase	GPI	0	0	0	0	0	0	297	0
Cornifin-B	SPRR1B	0	0	0	0	0	0	298	0
Clathrin heavy chain;Clathrin he	CLTC	0	0	0	0	0	0	299	0
Neutrophil gelatinase-associated	LCN2	0	0	0	0	0	0	300	0
Protein S100-P	S100P	0	0	0	0	0	0	302	0
60S ribosomal protein L18	RPL18	0	0	0	0	0	0	303	0
ATP synthase subunit alpha, mit	ATP5A1	0	0	0	0	0	0	304	0
Ribonuclease inhibitor	RNH1	0	0	0	0	0	0	305	0
14-3-3 protein gamma;14-3-3 pr	YWHAG	0	0	0	0	0	0	306	0
Plasminogen activator inhibitor 1	SERBP1	0	0	0	0	0	0	307	0

Antileukoproteinase	SLPI	82	104	97	0	109	146	0	0
Dihydropyrimidinase-related pro	DPYSL3;CRMP	0	0	0	143	0	170	0	0
Signal peptide, CUB and EGF-lil	SCUBE2	194	0	166	183	179	193	0	0
Attractin	ATRN	0	0	0	0	0	206	0	0
Dermcidin;Survival-promoting pe	DCD	161	0	0	0	0	207	0	0
Asporin	ASPN	196	154	189	0	0	208	0	0
Bone morphogenetic protein 1	BMP1	0	0	0	0	0	211	0	0
Myosin light polypeptide 6	MYL6	0	0	197	0	181	0	0	0
Ig heavy variable 1-69-2	IGHV1-69-2	0	0	0	0	183	0	0	0
Bactericidal permeability-increas	BPI	0	0	0	0	184	0	0	0
Insulin-like growth factor I	IGF1	212	0	0	0	186	0	0	0
Xin actin-binding repeat-contain	XIRP2	0	0	0	0	188	0	0	0
Tissue factor pathway inhibitor	TFPI	173	0	0	0	190	0	0	0
Deoxyribonuclease gamma;Deo	DNASE1L3	0	0	196	0	200	0	0	0
Prolactin-inducible protein	PIP	177	0	190	190	202	0	0	0
Hepatic triacylglycerol lipase	LIPC	0	0	0	0	206	0	0	0
Zymogen granule protein 16 hor	ZG16B	0	0	0	197	0	0	0	0
Ig heavy variable 6-1	IGHV6-1	0	0	0	205	0	0	0	0
Desmocollin-1	DSC1	152	0	0	207	0	0	0	0
Alpha-amylase 2B;Pancreatic al	AMY2B;AMY2A	0	0	0	209	0	0	0	0
T-complex protein 1 subunit gan	CCT3	0	0	179	0	0	0	0	0
Complement C1q tumor necrosi	C1QTNF3-AMA	179	155	181	0	0	0	0	0
Multimerin-1;Platelet glycoprotei	MMRN1	0	0	184	0	0	0	0	0
Angiopoietin-related protein 3	ANGPTL3	199	0	185	0	0	0	0	0
Insulin-like growth factor II;Insuli	IGF2	190	0	192	0	0	0	0	0
Olfactomedin-like protein 3	OLFML3	189	0	195	0	0	0	0	0
Serum deprivation-response pro	SDPR	0	0	198	0	0	0	0	0
Collectin-11	COLEC11	217	0	205	0	0	0	0	0
Ig light chain variable 3-10	IGLV3-10	0	141	0	0	0	0	0	0
Hemoglobin subunit delta	HBD	114	0	0	0	0	0	0	0
Carbonic anhydrase 1	CA1	140	0	0	0	0	0	0	0
Lipocalin-1	LCN1	143	0	0	0	0	0	0	0
Ig heavy variable 3OR16-32	IGHV3OR16-1	158	0	0	0	0	0	0	0
Catalase	CAT	171	0	0	0	0	0	0	0
Arginase-1	ARG1	181	0	0	0	0	0	0	0
Myeloperoxidase;Myeloperoxida	MPO	185	0	0	0	0	0	0	0
Peroxiredoxin-2	PRDX2	187	0	0	0	0	0	0	0
Prostaglandin-H2 D-isomerase	PTGDS	201	0	0	0	0	0	0	0
Ig heavy variable 3-35	IGHV3-35	205	0	0	0	0	0	0	0
Bleomycin hydrolase	BLMH	210	0	0	0	0	0	0	0
Corneodesmosin	CDSN	211	0	0	0	0	0	0	0
Serpin B12	SERPINB12	214	0	0	0	0	0	0	0
Alcohol dehydrogenase 4	ADH4	215	0	0	0	0	0	0	0
Ig lambda chain V-II region TOG	IGLV2-14	222	0	0	0	0	0	0	0
Keratinocyte proline-rich protein	KPRP	224	0	0	0	0	0	0	0
Fructose-1,6-bisphosphatase 1	FBP1	225	0	0	0	0	0	0	0
Beta-galactoside alpha-2,6-sialy	ST6GAL1	227	0	0	0	0	0	0	0
Collagen alpha-1(XVIII) chain;Er	COL18A1	229	0	0	0	0	0	0	0
Ig lambda-6 chain C region	IGLC6	231	0	0	0	0	0	0	0
Hepatocyte growth factor-like pr	MST1	232	0	0	0	0	0	0	0
Ig kappa chain variable 1D-13	IGKV1D-13	233	0	0	0	0	0	0	0
Disks large homolog 2	DLG2	237	0	0	0	0	0	0	0
Fatty acid-binding protein, adipo	FABP4	239	0	0	0	0	0	0	0
Mitochondrial calcium uniporter	MCUR1	240	0	0	0	0	0	0	0
Ig heavy variable 3-38	IGHV3-38	241	0	0	0	0	0	0	0
Filamin-A	FLNA	242	0	0	0	0	0	0	0
Plastin-2	LCP1	243	0	0	0	0	0	0	0
Ig kappa chain V-I region HK10	IGKV1D-16	244	0	0	0	0	0	0	0

(orange)	significant increase from 1min to 360min
(blue)	significant decrease from 1min to 360min
(green)	significant increase with high flow on same material
(grey)	significant increase on PET material with same flow rate
