# Supplementary material for: Comparative study of flow rate- and material-dependent human plasma protein adsorption on oxygenator membranes and heat exchanger materials
Source: Front Cardiovasc Med. 2025 Jun 17;12:1578538. doi: 10.3389/fcvm.2025.1578538 (PMC12211862; doi:10.3389/fcvm.2025.1578538)
Supplement: Supplementary file 6 [file Table4.pdf]

Supplementary Table 4: Complete list of all measured proteins, sorted by frequency on the heparin-coated PET membrane with a flow rate of 0.2 l/min at the indivdual time points after plasma contact (1-360min). Colored fields show significant changes (over time, between materials, or flow rates) according to the legend below.

		# of most abundance desorbed from minature devices - PET 0.2 l/min							
**Protein names**	**Gene names**	**1 min**	**5 min**	**10 min**	**30 min**	**60 min**	**90 min**	**180 min**	**360 min**
Serum albumin	ALB	4	1	4	4	1	5	2	1
Apolipoprotein B-100;Apolipopro	APOB	6	5	6	5	5	4	1	2
Fibrinogen alpha chain;Fibrinope	FGA	1	2	2	1	2	1	3	3
Fibrinogen beta chain;Fibrinope	FGB	2	3	1	2	3	2	4	4
Fibrinogen gamma chain	FGG	3	4	3	3	4	3	5	5
Complement C3;Complement C	C3	9	9	10	8	7	9	7	6
Apolipoprotein E	APOE	8	8	8	7	8	7	8	7
Antithrombin-III	SERPINC1	7	6	7	6	6	6	6	8
Fibronectin;Anastellin;Ugl-Y1;Ug	FN1	5	7	5	10	9	8	9	9
Serotransferrin	TF	10	10	14	13	10	21	16	10
Clusterin;Clusterin beta chain;Cl	CLU	41	40	33	23	25	13	15	11
Ficolin-2	FCN2	14	17	11	11	11	11	12	12
Ig gamma-1 chain C region	IGHG1	13	11	13	14	13	20	14	13
Apolipoprotein A-I;Proapolipopro	APOA1	15	15	16	15	16	14	17	14
Apolipoprotein(a)	LPA	26	26	24	21	17	12	13	15
Alpha-2-macroglobulin	A2M	18	13	19	19	14	22	22	16
Alpha-1-antitrypsin;Short peptide	SERPINA1	19	16	20	20	18	25	24	17
Lipopolysaccharide-binding prote	LBP	11	12	12	9	12	10	10	18
Inter-alpha-trypsin inhibitor heav	ITIH4	43	38	36	27	28	19	11	19
Ig mu chain C region	IGHM	21	19	18	17	19	16	20	20
Haptoglobin;Haptoglobin alpha c	HP	23	20	23	24	26	32	30	21
Hyaluronan-binding protein 2;Hy	HABP2	91	75	87	67	42	52	28	22
Plasma serine protease inhibitor	SERPINA5	44	46	44	40	33	36	26	23
Complement C1q subcomponen	C1QB	16	18	15	16	20	15	18	24
Complement C1q subcomponen	C1QC	17	22	17	18	22	17	19	25
Complement C4-A;Complement	C4A	30	25	26	25	24	24	23	26
Ig kappa chain C region	IGKC	22	21	21	22	23	23	25	27
Angiogenin	ANG	12	14	9	12	15	18	21	28
Ig alpha-1 chain C region	IGHA1	28	23	28	29	27	33	27	29
Vitronectin;Vitronectin V65 subu	VTN	32	36	30	30	31	28	33	30
Cholesteryl ester transfer protei	CETP	0	126	116	75	67	31	52	31
Complement C1q subcomponen	C1QA	24	33	25	26	30	26	29	32
Hemopexin	HPX	36	27	39	38	37	63	40	33
Immunoglobulin lambda-like poly	IGLL5;IGLC1	33	30	32	32	32	34	35	34
Ig gamma-2 chain C region	IGHG2	38	34	38	36	40	38	38	35
Inter-alpha-trypsin inhibitor heav	ITIH2	45	44	49	45	43	43	39	36
Coagulation factor XI;Coagulatio	F11	20	24	22	28	29	27	32	37
Ig gamma-3 chain C region	IGHG3	35	37	34	35	36	35	41	38
Alpha-1-acid glycoprotein 1	ORM1	69	49	67	78	54	104	78	39
Apolipoprotein A-IV	APOA4	50	50	45	44	51	45	36	40
Ceruloplasmin	CP	46	41	48	50	44	85	51	41
Plasminogen;Plasmin heavy cha	PLG	39	42	42	42	41	39	42	42
Vitamin D-binding protein	GC	47	43	53	47	46	92	46	43
Complement factor H	CFH	40	39	41	39	21	37	37	44
Serum amyloid P-component;Se	APCS	77	72	79	60	45	48	44	45
Alpha-1-antichymotrypsin;Alpha-	SERPINA3	52	48	63	52	49	64	56	46
Complement component C9;Cor	C9	109	97	100	92	88	78	48	47
Ig gamma-4 chain C region	IGHG4	58	56	76	61	60	105	76	48
Kininogen-1;Kininogen-1 heavy	KNG1	42	47	47	48	47	62	45	49
Complement C5;Complement C	C5	86	83	94	82	71	74	65	50
Inter-alpha-trypsin inhibitor heav	ITIH1	64	58	71	74	52	91	64	51
Alpha-2-HS-glycoprotein;Alpha-2	AHSG	66	54	70	65	59	89	61	52
C4b-binding protein alpha chain	C4BPA	60	61	54	49	39	50	60	53
Prothrombin;Activation peptide fr	F2	54	52	52	51	50	70	54	54
Complement factor B;Compleme	CFB	55	51	72	66	55	77	53	55
Apolipoprotein A-II;Proapolipopro	APOA2	62	68	61	59	62	59	62	56
Complement C1s subcomponen	C1S	37	35	37	33	38	29	34	57
Complement C1r subcomponent	C1R	31	28	31	31	34	30	31	58
Apolipoprotein C-II;Proapolipopro	APOC4-APOC2	93	79	90	64	69	47	49	59
Alpha-2-antiplasmin	SERPINF2	49	55	46	46	53	57	58	60
Angiotensinogen;Angiotensin-1;A	AGT	59	59	64	58	64	69	47	61
Plasma protease C1 inhibitor	SERPING1	68	66	69	69	58	88	83	62
Apolipoprotein D	APOD	84	86	95	77	75	61	55	63
Complement factor H-related pro	CFHR5	151	169	143	132	98	100	116	64
Beta-2-glycoprotein 1	APOH	65	57	85	85	76	106	101	65
Insulin-like growth factor-binding	IGFBP3	71	70	66	68	68	54	57	66
Ig heavy chain V-III region BUT	IGHV3-74;IGHV	81	78	83	76	74	68	87	67

Transthyretin	TTR	78	67	78	80	66	96	74	68
Lysozyme C;Lysozyme	LYZ	25	31	35	34	35	40	50	69
Alpha-1B-glycoprotein	A1BG	79	65	98	94	72	135	93	70
Mannan-binding lectin serine pro	MASP2	83	91	75	57	73	60	71	71
Hemoglobin subunit beta;LVV-he	HBB	89	88	60	71	83	87	85	72
Ficolin-3	FCN3	48	71	43	43	57	42	73	73
Heparin cofactor 2	SERPIND1	87	81	89	90	92	81	69	74
Serum paraoxonase/arylesterase	PON1	92	85	88	87	81	75	43	75
Phospholipid transfer protein	PLTP	144	151	163	117	129	71	86	76
Actin, cytoplasmic 1;Actin, cytop	ACTB	153	159	153	93	114	86	114	77
Apolipoprotein C-I;Truncated apo	APOC1	56	60	57	56	77	49	68	78
Apolipoprotein C-III	APOC3	85	76	77	73	85	58	82	79
Ig heavy variable 3-72	IGHV3-72	82	77	80	86	87	66	77	80
Ribonuclease 4	RNASE4	34	45	40	41	48	46	59	81
Histidine-rich glycoprotein	HRG	72	69	82	81	63	82	63	82
Apolipoprotein L1	APOL1	124	119	119	115	116	84	72	83
Retinoic acid receptor responder	RARRES2	53	74	62	62	61	53	81	84
Serum amyloid A-4 protein	SAA2-SAA4;SA	102	87	93	83	89	76	79	85
CD5 antigen-like	CD5L	96	100	97	89	90	83	99	86
Mannan-binding lectin serine pro	MASP1	73	89	56	72	70	73	88	87
von Willebrand factor;von Willeb	VWF	27	29	29	84	103	95	67	88
Leukocyte cell-derived chemotax	LECT2	51	53	50	53	65	55	75	89
Complement component C7	C7	131	140	132	147	121	133	92	90
Protein AMBP;Alpha-1-microglob	AMBP	76	84	84	91	84	93	96	91
Ig kappa chain V-II region FR	IGKV2D-28	95	90	102	106	105	123	113	92
Matrix Gla protein	MGP	115	133	0	99	94	159	109	93
Complement component C8 beta	C8B	122	144	151	142	122	128	91	94
Complement C4-B;Complement	C4B	137	110	121	120	111	115	104	95
Chondroadherin	CHAD	61	62	55	54	79	51	80	96
Complement factor H-related pro	CFHR1	114	108	117	107	99	102	90	97
Apolipoprotein A-V	APOA5	159	148	169	156	158	119	134	98
Complement component C6	C6	127	132	157	155	128	125	103	99
Ig kappa chain V-III region B6	IGKV3-20	130	104	110	125	106	0	123	100
Ig kappa chain V-I region AU;Ig kappa chain V-I		105	98	103	102	110	142	106	101
Proteoglycan 4;Proteoglycan 4 C	PRG4	29	32	27	37	56	41	66	102
Stromal cell-derived factor 1;SDF	CXCL12	97	105	106	95	93	80	94	103
Cadherin-1;E-Cad/CTF1;E-Cad/	CDH1	67	63	58	55	82	56	89	104
Alpha-1-antitrypsin;Short peptide	SERPINA1	110	109	128	101	96	143	122	105
Ig heavy variable 3-15	IGHV3-15	150	142	112	111	115	111	102	106
Glyceraldehyde-3-phosphate de	GAPDH	126	80	114	113	130	99	143	107
Insulin-like growth factor-binding	IGFBP5	63	73	59	63	78	44	95	108
Hemoglobin subunit alpha	HBA1;HBA2	112	113	81	98	107	109	100	109
Ribonuclease pancreatic	RNASE1	57	64	51	79	80	79	84	110
Ig kappa chain V-IV region	IGKV4-1	101	96	92	96	95	117	136	111
Zinc-alpha-2-glycoprotein	AZGP1	119	103	136	129	112	189	147	112
Glutathione peroxidase;Glutathio	GPX3	0	185	191	189	163	124	97	113
Immunoglobulin J chain	IGJ;JCHAIN	107	112	107	100	104	97	118	114
Afamin	AFM	103	95	122	114	102	164	140	115
Alpha-1-acid glycoprotein 2	ORM2	142	94	127	175	145	0	128	116
Complement component C8 alph	C8A	120	131	162	141	138	122	115	117
Histone H4	HIST1H4A	0	0	172	121	154	127	132	118
Coagulation factor XIII A chain	F13A1	70	82	65	70	97	72	144	119
Haptoglobin-related protein	HPR	117	129	120	116	120	130	110	120
Apolipoprotein M	APOM	155	143	141	128	135	144	112	121
Complement component C8 gam	C8G	140	138	138	134	132	136	117	122
Corticosteroid-binding globulin	SERPINA6	141	121	158	161	144	157	129	123
Fibulin-1	FBLN1	156	174	190	140	0	116	70	124
Carboxypeptidase N catalytic ch	CPN1	134	117	105	110	117	113	119	125
Lactotransferrin;Lactoferricin-H;K	LTF	94	102	96	105	119	90	130	126
N-acetylmuramoyl-L-alanine ami	PGLYRP2	132	130	142	151	139	177	133	127
Ig heavy variable 3-49	IGHV3-49	0	125	188	0	150	138	142	128
Kallistatin	SERPINA4	125	139	182	154	148	141	108	129
Inter-alpha-trypsin inhibitor heav	ITIH3	0	166	180	190	173	186	150	130
Tetranectin	CLEC3B	108	115	108	112	101	120	107	131
Properdin	CFP	160	0	0	109	86	98	105	132
Extracellular matrix protein 1	ECM1	80	92	74	97	126	101	152	133
Retinol-binding protein 4;Plasma	RBP4	111	106	134	133	108	0	127	134
Vitamin K-dependent protein S	PROS1	147	135	149	146	100	140	141	135
Ig heavy chain V-II region NEWM	IGHV4-61	146	162	123	0	142	129	155	136

Apolipoprotein C-IV	APOC4	154	156	145	131	133	132	111	137
Monocyte differentiation antigen	CD14	167	154	137	130	147	112	124	138
Insulin-like growth factor-binding	IGFALS	128	122	140	138	140	161	149	139
Prenylcysteine oxidase 1	PCYOX1	169	175	187	167	161	134	146	140
Plasma kallikrein;Plasma kallikre	KLKB1	100	136	109	124	123	196	153	141
Coagulation factor X;Factor X lig	F10	0	197	174	185	176	169	167	142
Pregnancy zone protein	PZP	116	128	99	123	124	107	125	143
Antileukoproteinase	SLPI	90	101	91	108	113	103	137	144
Coagulation factor V;Coagulation	F5	118	124	101	122	118	94	98	145
Tsukushin	TSKU	0	0	0	0	0	156	121	146
Ig lambda chain V-III region SH		148	0	139	143	164	126	161	147
Leucine-rich alpha-2-glycoprotei	LRG1	0	150	167	0	165	0	0	148
Carboxypeptidase N subunit 2	CPN2	135	137	148	152	149	148	151	149
Complement factor D	CFD	123	155	126	145	134	151	138	150
Pigment epithelium-derived facto	SERPINF1	149	146	179	176	162	179	165	151
C-reactive protein;C-reactive pro	CRP	0	0	184	182	186	165	126	152
Histone H2A type 1-J;Histone H2	HIST1H2AJ;HI	166	0	175	119	152	110	154	153
Procollagen C-endopeptidase en	PCOLCE	136	141	124	144	143	139	131	154
Coagulation factor XII;Coagulatio	F12	113	116	113	126	109	180	162	155
Neutrophil defensin 3;HP 3-56;N	DEFA3;DEFA1	0	0	0	0	159	137	168	156
Ig heavy variable 1-18	IGHV1-18	0	0	0	0	0	0	0	157
Ig heavy variabel 3OR16-9	IGHV3OR16-9	0	0	146	158	0	155	0	158
Ig heavy chain V-III region CAM;	IGHV3-23	133	0	0	0	160	0	158	159
Ig kappa chain V-III region VG	IGKV3D-11	0	0	133	0	137	0	166	160
Complement factor I;Complemen	CFI	161	134	170	174	181	0	177	161
Inter-alpha-trypsin inhibitor heav	ITIH4	0	0	0	0	0	65	0	162
Ig heavy variable 5-51	IGHV5-51	138	147	156	148	151	153	156	163
Coagulation factor XIII B chain	F13B	99	111	111	118	146	118	178	164
Ig lambda chain V-I region HA	IGLV1-47	0	158	159	170	0	175	184	165
Thrombospondin-4	THBS4	88	93	86	104	125	108	145	166
Procollagen C-endopeptidase en	PCOLCE2	139	161	135	165	167	145	148	167
Ig lambda chain V-IV region Hil;Ig lambda chain		0	149	0	150	153	0	120	168
Myosin-9	MYH9	129	120	104	103	127	67	157	169
Lumican	LUM	0	181	186	0	177	0	0	170
Histone H2B type 1-L;Histone H2	HIST1H2BL;HI	170	0	150	136	168	114	160	171
Band 3 anion transport protein	SLC4A1	0	0	0	0	206	166	212	172
Dihydropyrimidinase-related prot	DPYSL3;CRMP	74	0	0	0	0	0	0	173
Thyroxine-binding globulin	SERPINA7	0	178	166	0	185	0	182	174
Histone H3;Histone H3.3C;Histo	HIST2H3PS2;H	0	195	0	149	0	158	0	175
Pleckstrin	PLEK	121	127	115	127	131	121	159	176
EGF-containing fibulin-like extra	EFEMP1	177	0	0	0	0	171	172	177
Ig kappa chain V-I region Daudi;	IGKV1-6;IGKV	168	0	164	177	169	0	209	178
Serum amyloid A-2 protein	SAA2	0	0	0	0	0	0	0	179
Nephronectin	NPNT	152	145	125	163	170	131	170	180
C-C motif chemokine 14;HCC-1(	CCL14	106	118	118	137	141	146	173	181
Phosphatidylinositol-glycan-spec	GPLD1	0	198	193	0	193	0	197	182
Carboxypeptidase B2	CPB2	163	180	0	0	189	0	176	183
Cathelicidin antimicrobial peptide	CAMP	143	160	131	135	155	162	174	184
Sex hormone-binding globulin	SHBG	179	191	173	181	0	0	189	185
Protein Z-dependent protease in	SERPINA10	0	0	0	0	0	0	200	186
Selenoprotein P	SEPP1	0	189	0	0	188	182	181	187
Phospholipase A2, membrane as	PLA2G2A	158	0	161	166	166	163	0	188
Platelet factor 4;Platelet factor 4,	PF4;PF4V1	176	172	152	159	172	150	171	189
Ectonucleotide pyrophosphatase	ENPP2	164	212	144	173	157	174	163	190
Eosinophil cationic protein	RNASE3	0	167	178	168	183	160	179	191
Collagen alpha-3(VI) chain	COL6A3	0	0	0	0	0	190	183	192
Elongation factor 1-alpha 1;Puta	EEF1A1;EEF1A	0	173	183	184	171	154	188	193
Bactericidal permeability-increas	BPI	0	177	0	0	179	147	190	194
Lipoprotein lipase	LPL	181	200	165	178	194	178	205	195
Ig lambda variable 8-61	IGLV8-61	0	0	154	0	0	167	0	196
Hepatocyte growth factor activat	HGFAC	75	170	73	88	91	0	215	197
Protein S100-A9	S100A9	175	204	0	157	198	0	192	198
Attractin	ATRN	0	0	192	0	197	0	0	199
Galectin-3-binding protein	LGALS3BP	178	210	0	0	203	0	0	200
Serum amyloid A-1 protein;Amyl	SAA1	0	0	0	0	0	0	186	201
GTP-binding nuclear protein Ran	RAN	0	0	0	188	0	168	0	202
Fermitin family homolog 3	FERMT3	0	0	0	0	0	0	0	203
Fetuin-B	FETUB	0	0	0	0	0	0	194	204
Phosphatidylcholine-sterol acyltr	LCAT	0	0	0	0	0	181	0	205

Pyruvate kinase PKM;Pyruvate k	PKM	0	0	0	0	0	0	0	206
C4b-binding protein beta chain	C4BPB	0	0	0	0	200	0	0	207
Ig kappa variable 1-27	IGKV1-27	172	0	171	0	191	193	0	208
Biotinidase	BTD	0	0	0	0	201	0	0	209
Deoxyribonuclease gamma;Deo:	DNASE1L3	0	207	168	160	195	172	195	210
Fructose-1,6-bisphosphatase 1	FBP1	0	0	189	0	190	188	0	211
78 kDa glucose-regulated protei	HSPA5	0	0	0	0	0	0	0	212
Sulfhydryl oxidase 1	QSOX1	0	0	0	0	204	0	169	213
Transforming growth factor-beta-	TGFBI	0	0	0	0	0	184	180	214
Apolipoprotein F	APOF	0	0	0	0	205	195	208	215
Alcohol dehydrogenase 1B	HEL-S-117;AD	0	0	0	0	0	0	0	216
Complement C2;Complement C:	C2	0	218	0	0	0	0	0	217
ATP synthase subunit beta;ATP	ATP5B	0	0	0	0	0	0	0	218
Protein S100-A7;Protein S100-A	S100A7;S100A	165	216	0	0	0	0	0	219
Ig kappa chain V-I region BAN	IGKV1-16 BAN	0	0	0	0	0	0	0	220
Platelet-activating factor acetylh	PLA2G7	0	0	0	0	0	0	0	221
Hepatic triacylglycerol lipase	LIPC	0	0	0	0	0	191	0	222
Putative uncharacterized protein	MYH16;MYH7;	0	0	0	0	0	0	0	223
Myosin regulatory light chain 12/	MYL12A;MYL1	0	0	0	0	0	0	0	224
Cathepsin G	CTSG	0	0	0	0	0	0	0	225
Ig heavy variable 3-73	IGHV3-73	0	0	0	0	0	0	135	0
Adipocyte plasma membrane-as	APMAP	0	0	0	0	0	0	139	0
C-C motif chemokine 18;CCL18(	CCL18	145	171	147	164	156	149	164	0
Ig lambda chain V-VI region AR		0	0	0	0	0	0	175	0
Kininogen-1;Kininogen-1 heavy	KNG1	104	0	68	139	136	0	185	0
Ezrin;Radixin	EZR;RDX	0	0	0	0	0	0	187	0
Alcohol dehydrogenase 4	ADH4	0	0	0	0	0	0	191	0
Ig lambda variable 1-36	IGLV1-36	0	0	0	0	0	0	193	0
Ig kappa variable 3D15	IGKV3D-15	0	0	0	0	0	0	196	0
Solute carrier family 2, facilitated	SLC2A1	0	0	0	0	0	0	198	0
Complement C1r subcomponent	C1RL	0	0	0	0	0	0	199	0
Collagen alpha-1(XVIII) chain;Er	COL18A1	174	0	0	0	175	0	201	0
BPI fold-containing family B mem	BPIFB1	0	0	0	0	0	0	202	0
Phosphatidate phosphatase LPII	LPIN2	0	0	0	0	0	0	203	0
Triosephosphate isomerase	TPI1	0	0	0	0	0	0	204	0
Proprotein convertase subtilisin/l	PCSK6	0	0	0	0	0	194	206	0
Ig kappa chain V-I region HK102	IGKV1-5	0	0	0	0	0	0	207	0
Cofilin-1	CFL1	0	0	0	0	0	0	210	0
Talin-1	TLN1	0	0	0	0	0	187	211	0
Peroxiredoxin-6	PRDX6	0	0	0	0	0	0	213	0
Phosphatidylinositol 5-phosphate	PIP4K2A;PIP4I	0	0	0	0	0	183	214	0
Protein disulfide-isomerase A5	PDIA5	0	0	0	0	0	0	216	0
Ig kappa variable 6-21	IGKV6-21	0	0	0	0	0	0	217	0
Myosin light polypeptide 6	MYL6	0	184	0	179	199	152	0	0
Polyubiquitin-C;Ubiquitin;Ubiquit	UBC;UBB;RPS	0	163	0	0	0	170	0	0
Signal peptide, CUB and EGF-lik	SCUBE2	0	0	160	153	196	173	0	0
Multimerin-1;Platelet glycoprotei	MMRN1	0	208	0	0	0	176	0	0
Insulin-like growth factor I	IGF1	0	203	0	0	0	185	0	0
Bone morphogenetic protein 1	BMP1	0	0	0	0	0	192	0	0
Ig delta chain C region	IGHD	0	188	0	187	174	0	0	0
Ig Heavy Variable 1-69-2	IGHV1-69-2	157	0	176	0	178	0	0	0
Coiled-coil domain-containing pr	CCDC40	0	0	0	0	180	0	0	0
Ig eavy Variable 1/OR15-1	IGHV1OR15-1	0	0	0	0	182	0	0	0
Coagulation factor IX;Coagulatio	F9	180	209	0	171	184	0	0	0
Asporin	ASPN	0	202	155	172	187	0	0	0
Tissue factor pathway inhibitor	TFPI	0	0	0	0	192	0	0	0
Insulin-like growth factor II;Insuli	IGF2	0	0	185	0	202	0	0	0
Prolactin-inducible protein	PIP	0	219	0	186	0	0	0	0
Complement C1q tumor necrosis	C1QTNF3-AMA	182	0	0	183	0	0	0	0
Serum deprivation-response pro	SDPR	0	0	0	180	0	0	0	0
Tenascin-X	TNXB	0	0	0	169	0	0	0	0
Protein S100-A8;Protein S100-A	S100A8	0	0	0	162	0	0	0	0
Ig lambda chain V-III region LOI		0	0	129	0	0	0	0	0
Ig Heavy Variable 6-1	IGHV6-1	0	0	130	0	0	0	0	0
Insulin-like growth factor-binding	IGFBP4	0	0	177	0	0	0	0	0
Caldesmon	CALD1	0	186	181	0	0	0	0	0
Caspase-14;Caspase-14 subuni	CASP14	0	99	0	0	0	0	0	0
Calmodulin-like protein 5	CALML5	0	107	0	0	0	0	0	0
Bleomycin hydrolase	BLMH	0	114	0	0	0	0	0	0

Desmoplakin	DSP	183	123	0	0	0	0	0	0
Cathepsin D;Cathepsin D light cl	CTSD	0	152	0	0	0	0	0	0
Desmoglein-1	DSG1	0	153	0	0	0	0	0	0
Arginase-1	ARG1	0	157	0	0	0	0	0	0
Catalase	CAT	0	164	0	0	0	0	0	0
Protein-glutamine gamma-glutan	TGM3	0	165	0	0	0	0	0	0
Desmocollin-1	DSC1	0	168	0	0	0	0	0	0
Annexin A2;Annexin;Putative an	ANXA2;ANXA2	0	176	0	0	0	0	0	0
Junction plakoglobin	JUP	0	179	0	0	0	0	0	0
Galectin-7	LGALS7	0	182	0	0	0	0	0	0
NADP-dependent malic enzyme	ME1	0	183	0	0	0	0	0	0
Suprabasin	SBSN	0	187	0	0	0	0	0	0
Ig heavy chain V-III region DOB	IGHV3-9 DOB	0	190	0	0	0	0	0	0
Puromycin-sensitive aminopeptic	NPEPPS	0	192	0	0	0	0	0	0
Lysosome-associated membrane	LAMP1	0	193	0	0	0	0	0	0
Fatty acid-binding protein, epider	FABP5	0	194	0	0	0	0	0	0
Peroxiredoxin-1	PRDX1	0	196	0	0	0	0	0	0
Alpha-2-macroglobulin-like prote	A2ML1	0	199	0	0	0	0	0	0
Tartrate-resistant acid phosphata	ACP5	0	201	0	0	0	0	0	0
Malate dehydrogenase, mitochon	MDH2	0	205	0	0	0	0	0	0
Serpin B3	SERPINB3	0	206	0	0	0	0	0	0
Proteasome subunit alpha type-5	PSMA5	0	211	0	0	0	0	0	0
Coagulation factor VIII;Factor VI	F8	0	213	0	0	0	0	0	0
Proteasome subunit alpha type-7	PSMA7	0	214	0	0	0	0	0	0
Histidine ammonia-lyase	HAL	0	215	0	0	0	0	0	0
Cathepsin L2	CTSV	0	217	0	0	0	0	0	0
Carboxypeptidase;Lysosomal pr	CTSA	0	220	0	0	0	0	0	0
Protein-glutamine gamma-glutan	TGM1	0	221	0	0	0	0	0	0
Calpain-1 catalytic subunit	CAPN1	0	222	0	0	0	0	0	0
Voltage-dependent anion-selecti	VDAC2	0	223	0	0	0	0	0	0
Glycolipid transfer protein domai	GLTPD2	0	224	0	0	0	0	0	0
Fibronectin;Anastellin;Ugl-Y1;Ug	FN1	98	0	0	0	0	0	0	0
Ig Lambda Variable 3-10	IGLV3-10	162	0	0	0	0	0	0	0
Lipocalin-1	LCN1	171	0	0	0	0	0	0	0
Dermcidin;Survival-promoting pe	DCD	173	0	0	0	0	0	0	0
		significant increase from 1min to 360min							
		significant decrease from 1min to 360min							
		significant increase with high flow on same material							
		significant increase on PET material with same flow rate							
